# Dynamic Cutting Force Prediction Model and Experimental Investigation of Ultrasonic Vibration-Assisted Sawing

**DOI:** 10.3390/mi17020152

**Published:** 2026-01-23

**Authors:** Yangyu Wang, Yao Wang, Pengcheng Ni, Shibiao Qu, Qiaoling Yuan, Hui Wang, Xiaojun Lei, Jianfeng Wang, Yizhi Wang

**Affiliations:** 1 College of Mechanical Engineering, Zhejiang University of Technology, Hangzhou 310014, China; 2 Key Laboratory of Special Purpose Equipment and Advanced Processing Technology, Ministry of Education, Zhejiang University of Technology, Hangzhou 310014, China; 3 Shaoxing Shangyu Hangxie Thermal Power Co., Ltd., Shaoxing 312369, China; 4 Department of Transportation, Zhejiang Industry Polytechnic College, Shaoxing 312369, China; 5 Shangshang Desheng Group Co., Lishui 323499, China

**Keywords:** ultrasonic vibration-assisted sawing, band sawing, dynamic cutting force, prediction model, sawing efficiency, wash-boarding phenomenon

## Abstract

In conventional band sawing, the long-span compression of the flexible saw blade often results in large fluctuations in cutting force, low cutting efficiency, and poor force predictability. To address these issues, this study investigates the dynamic cutting force modeling and experimental validation of ultrasonic vibration-assisted band sawing using 304 stainless steel as the workpiece material. Based on an analysis of the band sawing mechanism, an ultrasonic vibration-assisted approach is proposed to modify the contact conditions between the saw blade and the workpiece. A dynamic model of the saw blade is established using the string vibration equation, and a multi-tooth dynamic cutting force prediction model is further developed by incorporating variable cutting depth characteristics under ultrasonic vibration. Comparative experiments are conducted between conventional sawing and ultrasonic vibration-assisted sawing to validate the proposed model. At feed rates of 0.1–0.4 mm/s and preload values of 0.1–0.5 mm, the proposed model predicts dynamic cutting forces with good agreement to experimental results, achieving an average relative error of 5.44%. Under typical cutting conditions for difficult-to-machine materials, ultrasonic vibration-assisted sawing reduces the average cutting force and feed force by approximately 15% and 18%, respectively, while decreasing surface roughness along the feed direction by about 21%, thereby improving sawing efficiency and surface quality.

## 1. Introduction

Over the past decade, band sawing has emerged as an efficient and low-material-loss machining method, widely applied in wood processing, metal cutting, and other industrial sectors, with relatively mature process systems and equipment [1]. Compared with circular sawing and wire sawing, band sawing features a slender, flexible, and continuously moving blade, demonstrating strong process adaptability and economic efficiency when cutting large-sized or difficult-to-machine materials [2]. Consequently, band sawing has attracted sustained attention in modern manufacturing environments that demand high efficiency and versatile processing equipment. However, as workpiece complexity and quality requirements continue to increase, conventional band sawing still faces significant challenges in cutting force prediction and control, as well as in suppressing the wash-boarding phenomenon.

In conventional band sawing, the ratio of chip thickness to cutting speed is relatively small, resulting in the coexistence of multiple material removal mechanisms, such as cutting, scraping, and plowing, which easily induce cutting force fluctuations. Furthermore, the saw blade exhibits a typical unsupported thin-strip structure between the guide arms. The complex contact conditions and cutting mechanisms between the blade and the workpiece lead to uneven local material removal rates. Small elastic deformations of the saw blade further cause dynamic variations in the cutting parameters [3]. These multi-factor coupling effects result in pronounced unsteady characteristics during the band sawing process, which not only affect the surface accuracy but also increase the risk of microscopic damage on the workpiece surface. In addition, when cutting hard outer layers with a band saw equipped with spring-mounted teeth, the wash-boarding phenomenon manifests as distinct striations and parallel cracks, leading to increased surface roughness and reduced machining precision [4].

To address the issues caused by thin chip thickness and the coexistence of scraping and plowing, Ni and colleagues classified the load characteristics of band sawing into steady-state and dynamic components, and introduced the equivalent principle considering the simultaneous presence of tooth cutting and scraping for their analysis [5]. B. F. Lehmann conducted a stress analysis of the band sawing process and pointed out that when multiple teeth participate in cutting, the saw blade exhibits pronounced intermittent dynamic fluctuations [6]. Min Wan and colleagues proposed a variable cutting depth model in milling research, systematically analyzing the influence of chip thickness variations on cutting forces, which provides a useful reference for modeling band sawing and other processes involving variable cutting depths [7].

In terms of cutting force prediction and modeling, C. Andersson proposed a method to measure the single-tooth cutting force during band sawing and analyzed its relationship with initial positional errors, tool dynamic characteristics, and tooth wear [8]. Tae Jo Ko and colleagues introduced the concept of relative cutting pressure to address the issue of skewed tooth sawing, enabling an equivalent representation of the cutting parameters in skewed tooth sawing [9]. Ni and colleagues combined the single-tooth cutting force models for straight and skewed teeth to propose an equidistant multi-tooth composite cutting force prediction method for the saw blade [10]. Yucheng Li and colleagues analyzed the temporal evolution of cutting forces and stress distributions using the Johnson–Cook constitutive model. They also developed a cutting force prediction model for EVC machining of SiCp/Al based on three-phase friction (TPF) theory, in which the friction components at the tool–chip interface (TCI), tool–particle interface (TPI), and tool–matrix interface (TMI) were quantified and predicted [11]. Wan and colleagues developed a prediction model for variable-depth milling forces, with a particular focus on the exit stage of metal cutting [12]. These studies provide a theoretical basis for the dynamic cutting force modeling presented in this work.

To reduce cutting forces and improve surface quality, ultrasonic vibration-assisted machining has been extensively studied in turning, milling, and related processes. Existing studies indicate that ultrasonic excitation can reduce stochastic vibrations and enhance machining efficiency by increasing the tool’s instantaneous stiffness, modifying tool–workpiece contact conditions, and inducing intermittent cutting. In his doctoral dissertation, Bai Wei proposed a two-dimensional ultrasonic elliptical vibration-assisted turning method, in which a horn was used to apply vibrational excitation, effectively reducing the average cutting force during turning [13]. Abootorabi and colleagues developed a cutting force model for ultrasonic vibration-assisted milling by jointly considering the effects of cutting speed and workpiece vibration amplitude, thereby improving the model’s prediction stability and applicability [14]. Wang and colleagues demonstrated that ultrasonic vibration-assisted wire sawing of single-crystal silicon significantly reduced the saw-kerf damage depth [15]. In addition, Tobias and colleagues systematically investigated low-frequency lateral vibration-assisted band sawing under vibration frequencies below 1000 Hz. The results showed that, at specific sawing speeds, a lateral vibration of approximately 800 Hz can reduce the main cutting force to some extent, but may adversely affect surface quality [16].

In summary, substantial progress has been made in band-saw cutting force modeling, process parameter optimization, and vibration-assisted machining. However, systematic studies on ultrasonic vibration-assisted band sawing remain limited, particularly regarding a unified theoretical framework for saw-blade dynamics and dynamic cutting force prediction. To address this gap, this study proposes an ultrasonic vibration-assisted band sawing approach and develops a corresponding dynamic cutting force prediction model, which is validated through sawing experiments. The effects of ultrasonic vibration on sawing performance are systematically examined in terms of cutting mechanisms, force characteristics, and surface quality, providing a theoretical basis for process optimization and engineering applications.

## 2. Dynamic Cutting Force Modeling

### 2.1. Main Cutting Force Model

Metal band sawing is essentially a compound process combining feed motion and sawing motion. Under conventional band sawing conditions, the tooth tip moves along the workpiece surface at an inclination angle determined by the ratio of cutting speed to feed speed. Because the blade’s linear speed is much higher than the feed speed, this angle is typically small. Therefore, in conventional sawing, the actual cutting depth of a single tooth can be approximated as constant and taken as the average cutting depth between adjacent teeth [17].

**Figure 1:**
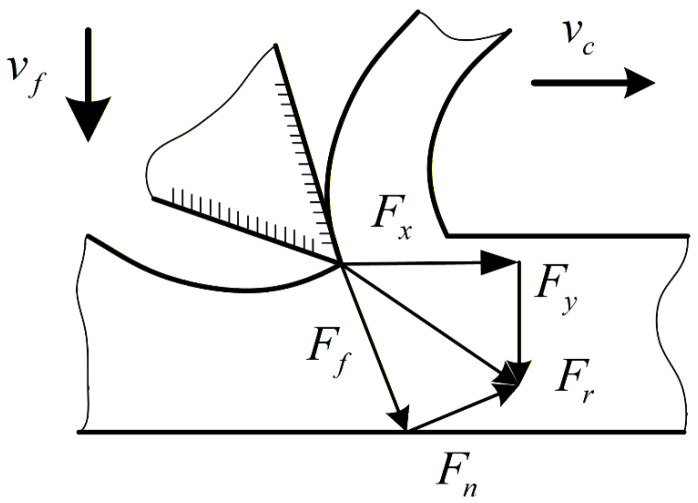
Schematic of short-duration variable-depth sawing by a single tooth.

This study develops a mechanical model for band sawing with variable cutting depth. The material removal force in single-tooth sawing is denoted by $F_{r}$, and the rake-face friction force by $F_{f}$. Based on these definitions, we analyze cutting-force characteristics under ultrasonic vibration-assisted band sawing and investigate the dynamic cutting force under variable cutting depth. As illustrated in Figure 1, $F_{r}$ is the material removal force generated by a single tooth during transient contact; $F_{f}$ is the friction force between the rake face and the chip; and $F_{n}$ is the normal compressive force exerted by the rake face on the chip. Here, $F_{x}$ and $F_{y}$ are the components of $F_{r}$ in the sawing and feed directions, respectively.

In the modeling, we focus on the single-tooth cutting force under the coupled action of the normal compressive force exerted by the rake face on the chip and the rake-face friction force. Accordingly, the single-tooth cutting force is expressed as:

(1) $$F_{d}=F_{x}=\lambda F_{r}$$

Here is a dimensionless proportional coefficient that quantifies the contribution of rake-face friction to the total cutting force.

Considering experimental measurability and engineering needs, this study first examines the main cutting force, $F_{x}$, in the sawing direction. By refining and extending the main cutting force model, we develop a cutting force prediction model for ultrasonic vibration-assisted band sawing and further evaluate how ultrasonic vibration alters the cutting force characteristics.

Based on the cutting characteristics of band sawing and an analysis of tooth geometry, Ko and Kim established a relationship between the material removal volume and the undeformed chip cross-sectional area [10]. Accordingly, a single-tooth main cutting force model dominated by rake-face material removal is proposed, as expressed by:

(2) $$F_{xi}=k_{t}{A_{i}}^{\beta }$$

where $k_{t}$ is the specific sawing pressure (N/mm^2^), $A_{i}$ is the undeformed chip cross-sectional area of the tooth i, and β is the (dimensionless) sawing exponent.

### 2.2. Variable-Depth Cutting Force Prediction Model for an Ultrasonically Excited Saw Blade

Metal band sawing is a coupled process involving both feed motion and sawing motion. In conventional band sawing, the tooth tip travels along the workpiece surface at an inclination angle governed by the cutting-to-feed speed ratio. Because the blade’s linear speed far exceeds the feed speed, the inclination angle is typically small. Accordingly, the actual cutting depth of an individual tooth can be approximated as constant, equal to the mean cutting depth between adjacent teeth [18]. Let p denote the tooth pitch (the spacing between adjacent teeth). The mean cutting depth, $d_{f0}$, is then given by Equation (3):

(3) $$d_{f0}=\bar{d_{fi}}=\frac{\sum_{i=1}^{n}d_{fi}}{n}=\frac{60pv_{f}}{1000v_{c}}$$

Under these conditions, the teeth cut continuously within each time interval, and the process can be approximated as quasi-steady. With ultrasonic vibration assistance, the cutting kinematics change substantially. With ultrasonic vibration assistance, the cutting kinematics change substantially. Ultrasonic vibration-assisted sawing increases the tooth’s instantaneous feed velocity, $v_{f}\left(t\right)$, thereby altering tooth–workpiece contact and the associated material removal mechanisms. In lateral ultrasonic vibration-assisted band sawing, an ultrasonic oscillation with a prescribed frequency and amplitude is superimposed along the feed direction on the blade’s axial motion. As a result, the originally continuous blade–workpiece contact is transformed into a high-frequency intermittent cutting process. Under ultrasonic excitation, the blade periodically engages with and disengages from the workpiece, enabling intermittent material removal.

**Figure 2:**
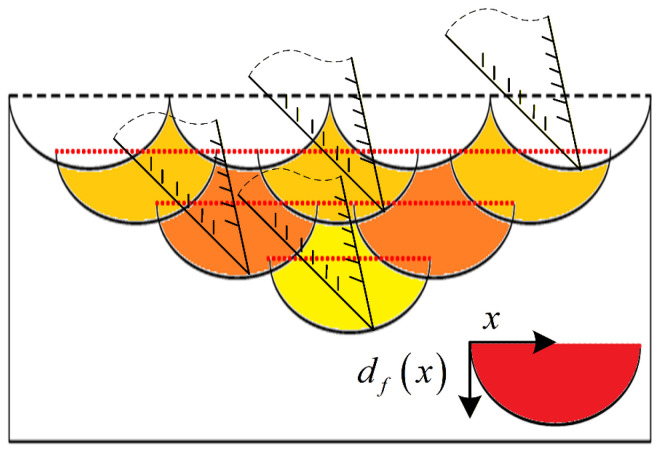
Schematic of the variable-depth cutting cross-section.

Unlike conventional sawing, where the mean cutting depth can be treated as a constant $d_{f0}$, ultrasonic vibration-assisted sawing exhibits a strongly time-varying instantaneous cutting depth within each excitation cycle, from initial tooth engagement to complete disengagement. The cutting cross-section varies with vibration phase, and its overall profile can be approximated as a locally sinusoidal function, as shown in Figure 2.

For the tension-dominated thin band saw blade, vibrations induced by tensile loading are analyzed using a string vibration model to describe the transverse response under ultrasonic excitation and to formulate the instantaneous cutting depth within a single vibration cycle. Based on the derived equivalent cutting cross-sectional area, a variable-depth dynamic cutting force prediction model incorporating ultrasonic vibration effects is established.

To establish the proposed theoretical model, the following assumptions are made based on the physical characteristics of the actual sawing system:

- Ideal string model:
  The band saw blade is modeled as a vibrating string subjected to high axial tension. Owing to the sufficiently large length-to-thickness ratio of the blade, bending stiffness is neglected, and axial tension is considered the dominant restoring force. Although neglecting bending stiffness may introduce minor discrepancies in higher-order vibration modes, the string model provides an accurate representation of the fundamental vibration behavior, which is dominant in the present study.
- Boundary conditions (pinned–sliding support):
  The system is modeled with a pinned support at one end and a sliding pinned support at the other end. This configuration accurately represents the constraints imposed by the guide arm system in practical band sawing machines, where one end is fixed by the guide block, while the other end is constrained by the ultrasonic excitation guide wheel. This arrangement allows axial tension adjustment while restricting transverse displacement.
- Linear transverse vibration assumption:
  The transverse vibration of the band saw blade is assumed to be of small amplitude, such that linear wave theory can be applied. To ensure the validity of the linear vibration model, the maximum transverse vibration amplitude is constrained to be less than 2% of the vibration span length.

**Figure 3:**
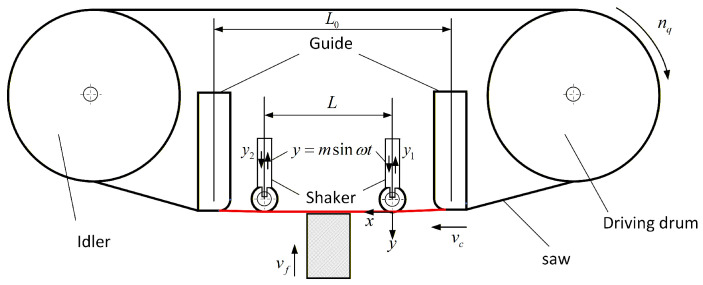
Schematic of the excitation method.

During high-speed band sawing, the blade predominantly exhibits high-frequency lateral vibrations. Imposing a controlled lateral ultrasonic vibration can suppress stochastic vibrations, thereby improving process stability and machining performance [16]. As shown in Figure 3, a blade-fixed coordinate system is established with the excitation contact point near the drive wheel as the origin; the sawing direction is defined as the x-axis, and the feed direction as the y-axis. The blade is assumed to be excited ultrasonically with frequency ω and amplitude m. After the excitation wheel is pressed down and a preload force is applied, the exciter remains in intimate contact with the blade during cutting, enabling effective control of the blade’s vibration state.

**Figure 4:**
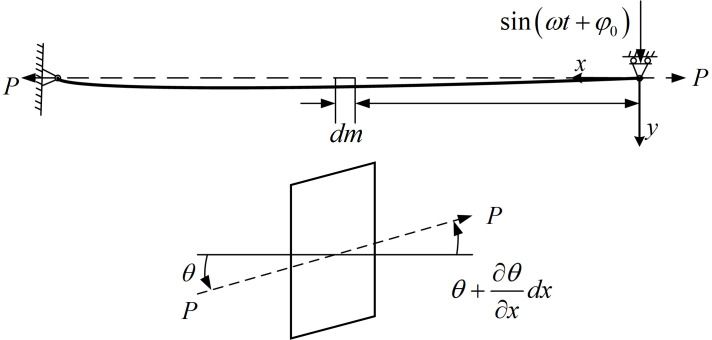
Principle of the string vibration equation.

We further analyze the saw blade segment between the guide arms, with length $L_{0}$, height $l_{0}$, and thickness $w_{0}$. During high-speed sawing, the blade predominantly vibrates perpendicular to the kerf. Introducing controlled ultrasonic vibration can suppress stochastic vibrations and improve process stability. Based on the modeling assumptions, the band saw blade is treated as a uniformly tensioned string, and its dynamic mechanical behavior is analyzed using string vibration theory, such as traveling-wave solutions. Owing to the sufficiently large length-to-thickness ratio of the blade, axial tension acting along the blade axis serves as the dominant restoring force, while bending stiffness can be neglected. Accordingly, the band saw blade is simplified as a vibrating string with a pinned support at one end and a sliding pinned support at the other. After establishing the coordinate system shown in Figure 4, the forced vibration of the band saw blade can be equivalently described as a continuous string system.

As reported in Ref. [19], Using a coordinate transformation, the transverse vibration equation of a string moving at a constant axial speed can be derived as:

(4) $$\frac{\partial^{2}y}{\partial t^{2}}+2v_{c}\frac{\partial^{2}y}{\partial x\partial t}-\left(c^{2}-{v_{c}}^{2}\right)\frac{\partial^{2}y}{\partial x^{2}}=0$$

where $y\left(x,t\right)$ denotes the transverse displacement of a point x on the moving string relative to its equilibrium position at time t; $v_{c}$ is the axial transport speed of the string; and c is the transverse wave speed for the corresponding stationary string. The transverse wave speed is given by $c=\sqrt{P/\rho S}$, where ρ is the blade material density, S is the mean cross-sectional area of the blade, and P is the blade tension. The upstream and downstream locations of the workpiece are imposed as boundary conditions in the string vibration model. The workpiece is centered between the guide arms with a spacing of $L_{0}$, and the distance between the two excitation locations is L. The applied ultrasonic excitation is $y=m\sin\left(\omega t\right)$. The boundary conditions at the excitation locations and the corresponding analytical solutions are summarized in Table 1.

Under lateral vibration excitation applied at the upstream and downstream positions, this study focuses on the cutting region (0,L). Given the variation in workpiece width, the cutting-depth function at x=L/2 is adopted as an approximation to represent the instantaneous cutting-depth evolution of the tooth during sawing.

When the cutting depth in the band sawing process is approximated as a time-dependent function at the center position of the workpiece, the spatial coordinate can be taken as x=L/2. Under this approximation, the dynamic response at the mid-span cutting point is investigated. The results indicate that, regardless of whether the ultrasonic excitation is applied upstream or downstream of the cutting zone, the moving band saw blade exhibits identical vibration behavior at the central cutting position.

Accordingly, by substituting x=L/2 into the analytical solutions of the forced vibration equations for upstream excitation $y_{1}\left(x,t\right)$ and downstream excitation $y_{2}\left(x,t\right)$, Under transverse ultrasonic excitation, the vibration response at the mid-span position is expressed as followed:

(5) $$y\left(\frac{L}{2},t\right)=y_{1}\left(\frac{L}{2},t\right)=y_{2}\left(\frac{L}{2},t\right)=-mi\frac{\sin\left[\omega \left(\frac{L_{0}}{2}\right)/c^{\prime }\right]}{\sin\left(\omega \frac{L+L_{0}}{2}/c^{\prime }\right)}\sin\left[\omega \left(t-\frac{L}{2v^{\prime }}\right)\right]$$

The analysis indicates that variations in the upstream and downstream positions of the ultrasonic vibration excitation have a negligible effect on the vibration response at the central cutting point of the moving band saw. This finding validates the consistency and reliability of the vibration model under different excitation positions. Furthermore, the results further confirm the applicability of the Maxwell-Betti reciprocal theorem within the symmetric structural system of the band saw.

According to Equation (5), during the vibration-assisted band saw cutting process, the motion of the central cutting point on the band saw under forced vibration can be approximated as simple harmonic motion. Under lateral ultrasonic excitation, the transverse vibration displacement at the central cutting point of the band saw can be simplified as:

(6) $$y_{\frac{L}{2}}\left(t\right)=KZ\sin\left(\omega t+\varphi_{0}\right)$$

where K is the amplitude amplification factor, and $\varphi_{0}=-\frac{L\omega }{2v^{\prime }}$.

Accordingly, the blade’s cutting-direction vibration velocity under vibration-assisted sawing is expressed as:

(7) $$v_{\frac{L}{2}}\left(t\right)=KZ\omega \cos\left(\omega t+\varphi_{0}\right)$$

where Z is the excitation amplitude, and ω is the excitation angular frequency.

When the ultrasonically excited blade cuts the workpiece, the complex blade–workpiece contact conditions prevent the cutting depth from being strictly synchronized with the vibration displacement amplitude. Accordingly, the effective cutting time of a tooth within a single vibration cycle is determined from the cutting-area conservation principle, yielding a time-dependent function for the cutting depth [20].

**Figure 5:**
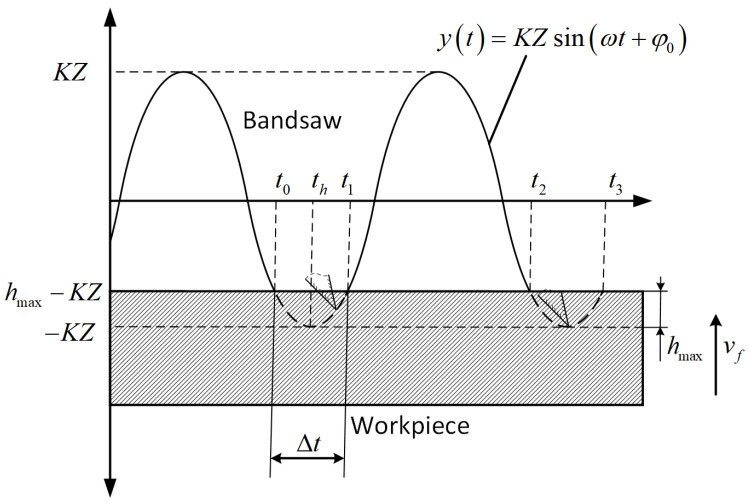
Analysis of variable-depth vibration-assisted sawing modes for a single band-saw tooth.

Figure 5 illustrates the variable-depth, vibration-assisted sawing process of a single band-saw tooth. Here, $t_{0}$, $t_{1}$, $t_{2}$, and $t_{3}$ denote the instants when the same tooth tip enters and exits the workpiece within a single vibration cycle. Let the maximum cutting depth within one vibration cycle be $h_{\max}$, and let $t_{h}$ denote the instant at which the cutting depth attains $h_{\max}$, satisfying $t_{0}<t_{h}<t_{1}$. Since the workpiece feed speed relative to the blade, $v_{f}$, is much smaller than the tooth’s instantaneous vibration velocity during vibration-assisted sawing, it follows from Figure 5 that, at the tooth-entry and tooth-exit instants, the cutting velocity has the same magnitude but opposite directions, i.e.,

(8) $$v_{\frac{L}{2}}\left(t_{0}\right)=-v_{\frac{L}{2}}\left(t_{1}\right)=v_{\frac{L}{2}}\left(t_{2}\right)=-v_{\frac{L}{2}}\left(t_{3}\right)$$

Let the effective cutting time within a single vibration cycle be $\Delta t=t_{1}-t_{0}=2(t_{h}-t_{0})$. Since the feed velocity $v_{f}$ is much smaller than the vibration velocity during vibration-assisted sawing, its influence can be neglected to a first approximation. Setting $v_{f}\approx 0$ and f=ω/2π yields:

(9) $$\left\{\begin{array}{l} -KZ=KZ\sin\left(2\pi ft_{h}+\varphi_{0}\right)\\ h_{\max}-KZ=KZ\sin\left(2\pi ft_{0}+\varphi_{0}\right) \end{array}\right.$$

Solving yields:

(10) $$\left\{\begin{array}{l} t_{h}=\frac{3}{4f}-\frac{\varphi_{0}}{2\pi f}\\ t_{0}=\frac{1}{2\pi f}\arcsin\left(\frac{h_{\max}}{KZ}-1\right)-\frac{\varphi_{0}}{2\pi f}\\ t_{1}=\frac{1}{\pi f}\left(\frac{3\pi -\varphi_{0}}{2}-\frac{1}{2}\arcsin\left(\frac{h_{\max}}{KZ}-1\right)\right) \end{array}\right.$$

Since the macroscopic workpiece feed velocity relative to the blade, $v_{f}$, remains unchanged during vibration-assisted sawing, the total material removal over a given time interval is conserved. Therefore, the maximum cutting depth must satisfy a cutting-area conservation condition. For vibration-assisted intermittent cutting with entry and exit instants $t_{0}$ and $t_{1}$, the maximum cutting depth $h_{\max}$ satisfies:

(11) $$\int_{t_{0}}^{t_{1}}h_{\max}-KZ-y\left(t\right)dt=v_{f}T$$

i.e.,

(12) $$\frac{\left(h_{\max}-KZ\right)\Delta t-v_{f}T}{KZ}=\int_{\frac{1}{2\pi f}\arcsin\left(\frac{h_{\max}}{KZ}-1\right)-\frac{\varphi_{0}}{2\pi f}}^{\frac{1}{\pi f}\left(\frac{3\pi -\varphi_{0}}{2}-\frac{1}{2}\arcsin\left(\frac{h_{\max}}{KZ}-1\right)\right)}\sin\left(wt+\varphi_{0}\right)dt$$

To characterize the intermittent cutting behavior in vibration-assisted sawing, a characteristic function $\Omega \left(t\right)$ is introduced to represent the cutting state, defined as:

(13) $$\Omega \left(t\right)=\left\{\begin{array}{l} 1,t_{0}+\frac{2n\pi }{\omega }<t<t_{1}+\frac{2n\pi }{\omega }\\ 0,t_{1}+\frac{2n\pi }{\omega }<t<t_{0}+\frac{2n\pi }{\omega } \end{array}\right.$$

Accordingly, the tooth’s actual cutting depth $df\left(t\right)$ during vibration-assisted sawing is expressed as:

(14) $$df\left(t\right)=y\left(\frac{L}{2},t\right)\cdot \Omega \left(t\right)=\left(h_{\max}-KZ-KZ\sin\left(\omega t+\varphi_{0}\right)\right)\cdot \Omega \left(t\right)$$

Hence, the undeformed chip cross-sectional area A can be expressed as:

(15) $$A=b_{l}df(t)$$

where $b_{l}$ is the tooth cutting width, with units of mm.

**Figure 6:**
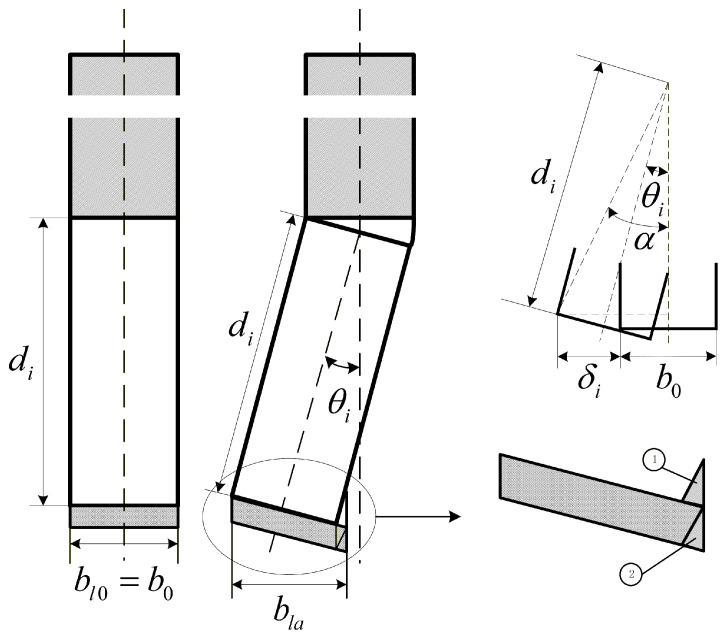
Optimized sawing cross-section.

Because ultrasonic vibration-assisted sawing involves a short-duration [10], variable-depth cutting mechanism, the conventional thin-chip thickness formulation cannot accurately capture this behavior. Accordingly, the cutting cross-section is modified as shown in Figure 6. The figure illustrates the cutting cross-sections of the straight tooth and the skewed tooth indexed by i. Here, $d_{i}$ denotes the tooth height, $\theta_{i}$ the skew angle of the i-th tooth, $\delta_{i}$ the skew width, and $b_{0}$ the tooth pitch; all teeth are assumed to share identical geometry. The cutting width is $b_{l0}$ for the straight tooth and $b_{li}$ for the skewed tooth.

For the skewed-tooth cross-section, an equivalent transformation that maps region (②) to region (①) is adopted to improve the accuracy of the cutting cross-sectional area calculation. In practical blade manufacturing, skewed teeth are typically specified by the skew width $\delta_{i}$. In the present theoretical analysis, however, the skew angle $\theta_{i}$ is used as the primary descriptor. Accordingly, the following functional relationship is introduced, where α is an auxiliary angular variable.

$$\left\{\begin{array}{l} \frac{\frac{1}{2}b_{0}+\delta_{i}}{\sqrt{{d_{i}}^{2}+\frac{1}{4}{b_{0}}^{2}}}=\sin\alpha \\ tg\left(\alpha -\theta_{i}\right)=\frac{\frac{1}{2}b_{0}}{d_{i}} \end{array}\right.$$

(16) $$\theta_{i}=arc\sin\left(\frac{\frac{1}{2}b_{0}+\delta_{i}}{\sqrt{{d_{i}}^{2}+\frac{1}{4}{b_{0}}^{2}}}\right)-tg^{-1}\left(\frac{\frac{1}{2}b_{0}}{d_{i}}\right)$$

As shown in Figure 6, when the cross-sectional overlap between adjacent teeth is neglected, the cutting cross-section width can be expressed as follows:

- Cutting width for straight teeth:
  $$b_{l0}=b_{0}$$
- Cutting width for skewed teeth:
  $$b_{la}=b_{0}+df_{i}\left(t\right)\sin\theta_{i}$$

The material removal cross-section function derived under the constant chip-thickness assumption is no longer applicable to ultrasonic vibration-assisted sawing. Therefore, the material removal function is further refined in this study. From the above expressions, the instantaneous cutting cross-sectional area of a single tooth is denoted by $A_{i}$. Neglecting cross-section overlap between adjacent teeth, the cutting area of the i-th straight tooth is $A_{0i}$, whereas that of the skewed tooth is $A_{ai}$.

(17) $$A_{i}=\left\{\begin{array}{l} A_{0i}=b_{0}df_{i}\left(t\right)\\ A_{ai}=\left(b_{0}+df_{i}\left(t\right)\sin\theta_{i}\right)df_{i}\left(t\right) \end{array}\right.$$

Owing to the time offset between the skewed and straight teeth at the same workpiece cross-section, the (i−1)-th tooth reaches the workpiece center section at $t_{i-1}$. When the blade subsequently reaches the same section at $t_{i}$, the corresponding cutting-layer depths are $df_{i-1}\left(t_{i-1}\right)$ for the (i−1)-th tooth and $df_{i}\left(t_{i}\right)$ for the i-th tooth. Accordingly, quantifying the overlap area becomes essential. To account for adjacent-tooth overlap, the intersection area $A_{si}$ is derived as follows.

**Figure 7:**
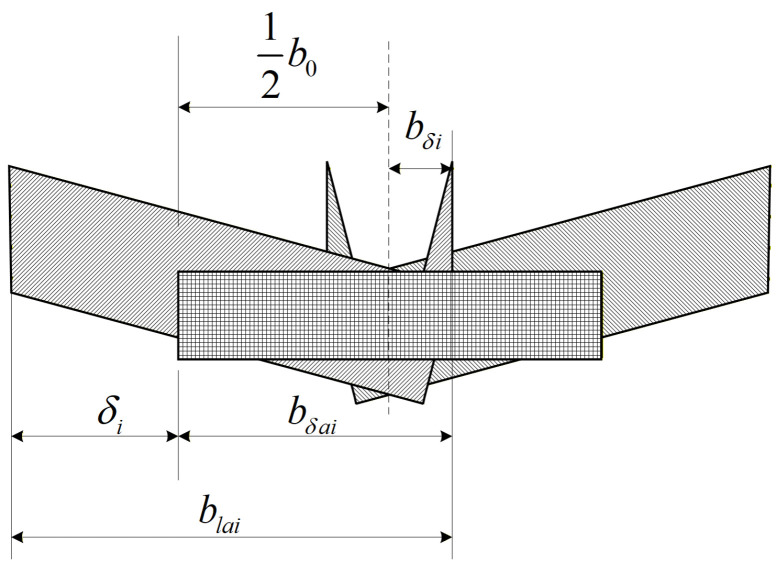
Schematic of the cutting cross-sections for straight teeth and left/right set teeth, illustrating the cross-sectional shapes along the cutting direction and the overlapping regions of different tooth profiles, with the overlap widths labeled as $b_{lai}$ and $b_{\delta ai}$. Shaded areas: Cutting cross-sections of different tooth profiles.

As a first step, the overlap width between adjacent teeth is identified. The overlap width for a skewed–straight tooth pair is denoted by $b_{sa,i}$, whereas that for two skewed teeth is denoted by $b_{sb,i}$. The corresponding geometric relations are given in Figure 7:

(18) $$\left\{\begin{array}{l} b_{\delta ai}=b_{la}-\delta_{i};\\ b_{\delta bi}=b_{\delta 1}+b_{\delta 2};\\ b_{\delta i}=b_{la}-\delta_{i}-\frac{1}{2}b_{0}. \end{array}\right.$$

Since intersections occur between straight and skewed teeth, the straight-tooth length can be neglected. Moreover, the equal-length skewed–skewed intersection can be treated as a special case of skewed–skewed intersection. Accordingly, three intersection cases are considered: straight–skewed, skewed–skewed, and unequal-length skewed–skewed. These cases cover all possible interactions between large and small teeth involving skewed teeth. Because the cross-sectional geometry in ultrasonic vibration-assisted sawing is complex, overlap cases are segmented according to the relationship between the preceding-tooth cutting-layer depth $d_{f,i-1}\left(t_{i-1}\right)$ and the succeeding-tooth cutting-layer depth $d_{f,i}\left(t_{i}\right)$. For convenience, a dimensionless parameter—the adjacent-tooth cutting-depth ratio, $\varepsilon_{df,i}$—is introduced as:

(19) $$\varepsilon_{dfi}=\frac{df_{i-1}\left(t_{i-1}\right)}{df_{i}\left(t_{i}\right)}$$

**Figure 8:**
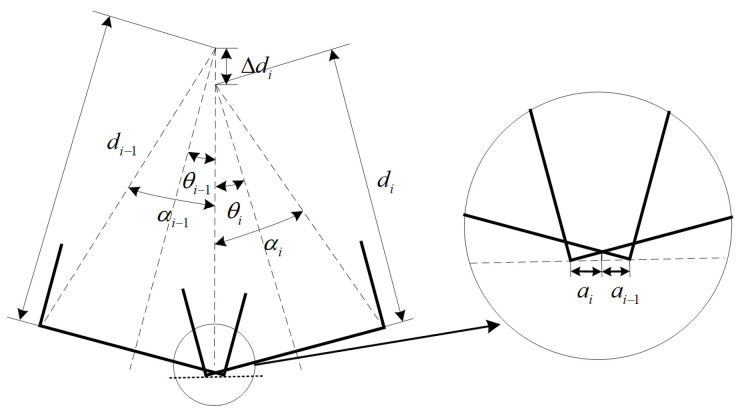
Auxiliary quantities for calculation.

In addition, for the skewed–skewed intersection case, the baseline overlap cross-section of adjacent skewed teeth is characterized by introducing the skewed-tooth intercept coefficient $a_{i}$ and the nominal tooth-length difference $\Delta d_{i}$. As shown in Figure 8, the relationship is expressed as:

$$\left\{\begin{array}{l} a_{i-1}\tan\theta_{i-1}=a_{i}\tan\theta_{i}\\ a_{i-1}+a_{i}=b_{0}\left(\cos\theta_{i-1}+\cos\theta_{i}\right)-\left(\sqrt{\frac{1}{4}{b_{0}}^{2}+{d_{i}}^{2}}\sin\alpha_{i}+\sqrt{\frac{1}{4}{b_{0}}^{2}+{d_{i-1}}^{2}}\sin\alpha_{i-1}\right) \end{array}\right.$$

(20) $$\Delta d_{i}=\cos\alpha_{i-1}\sqrt{\frac{1}{4}{b_{0}}^{2}+{d_{i-1}}^{2}}-\cos\alpha_{i}\sqrt{\frac{1}{4}{b_{0}}^{2}+{d_{i-1}}^{2}}+b_{0}\left(\sin\theta_{i-1}-\sin\theta_{i}\right)$$

**Figure 9:**
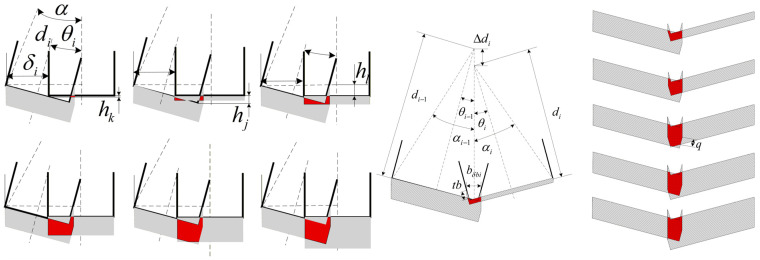
Interference cross-sections between adjacent teeth. Red areas: Overlap of cutting cross-sections of adjacent teeth.

Owing to the interchangeability of the overlap cross-sections between two teeth, a more detailed analysis of the overlap area is conducted, with particular emphasis on cross-section interference at the same section under ultrasonic excitation. In the cross-section analysis, the skewed-tooth cutting-layer depth $df_{i}\left(t_{i}\right)$ is selected as the reference. The straight-tooth cutting-layer depth $df_{i-1}\left(t_{i-1}\right)$ is then varied to investigate the resulting complex cross-section morphology and to examine the variable-depth behavior as $\varepsilon_{dfi}$ changes. As shown in Figure 9, the interference cross-section expressions are derived by considering both skewed–straight and skewed–skewed interactions between adjacent teeth. The resulting formulas are summarized in Table 2, and the corresponding parameter definitions are provided in Table 3.

In summary, after accounting for the adjacent-tooth overlap effect, the overlap cross-section $A_{\delta i}$ is expressed as:

(21) $$A_{\delta i}=\left\{\begin{array}{l} A_{\delta ai}\\ A_{\delta bi} \end{array}\right.$$

Accordingly, in variable-depth band sawing, the corrected single-tooth cutting cross-section ${A_{i}}^{\prime }$ is obtained by accounting for the overlap-induced cross-section error $A_{\delta i}$:

(22) $${A_{i}}^{\prime }=A_{i}-A_{\delta i}$$

Following Ni et al.’s analysis of specific cutting pressure, the specific sawing pressure $k_{t}$ in the sawing model is expressed as:

(23) $$k_{t}=k_{1}k_{2}k_{3}=2.8{\left(\frac{\varepsilon \%}{16\%}\right)}^{0.2}\sigma_{b}k_{p}f\left(t\right)k_{w}\lambda {v_{c}}^{\alpha }{v_{f}}^{\varepsilon }$$

In practical sawing, the specific sawing pressure $k_{t}$ varies with the feed and cutting speeds; therefore, $k_{t}$ differs across operating conditions. Its value can be identified via regression using experimental measurements of the cutting force and feed force [10]. From the above analysis, in vibration-assisted band sawing, the main cutting force associated with a single tooth can be simplified as a linear function of the undeformed cutting area:

(24) $$F_{xi}=k_{t}{A_{i}}^{\prime \beta }=k_{t}{A_{i}}^{\prime }+k_{\varepsilon }$$

here $k_{\varepsilon }$ is the offset.

When adjacent-tooth cross-section overlap and ultrasonic-excitation-induced variable chip thickness are considered, the main cutting force $F_{X0}$ during a time interval over which the single-tooth cutting state remains unchanged can be expressed as:

(25) $$F_{X0}=\sum_{i=m}^{n}F_{xi}$$

where n is the number of cutting teeth, i is the tooth index, and m denotes the first tooth currently engaged in cutting. When the cutting distance reaches the pitch between the n-th and (n−1)-th teeth, m updates accordingly, and the main cutting force is obtained by superposing the single-tooth forces from (m−1) to (n−1).

Because the kerf sidewall friction increases rapidly with cutting depth, the variable-depth ultrasonic vibration-assisted sawing process is examined in further detail. Following the work of P. V. Bayly et al. on intermittent milling, the tool motion can be represented by a delay differential equation with regenerative effects, which captures the influence of sidewall friction in a first approximation. Accordingly, the cutting process is simplified as a first-order inertial system [21]. Accordingly, a first-order inertial system is introduced to refine the cutting force model, and the resulting discretized difference equation is expressed as:

(26) $$F_{X}\left(n\right)=\alpha \cdot F_{X}\left(n-1\right)+\left(1-\alpha \right)\cdot F_{X0}\left(n\right)$$

where $F_{X0}\left(n\right)$ denotes the instantaneous cutting force at the current step without regenerative effects; $F_{X}\left(n\right)$ denotes the instantaneous cutting force at the current step with regenerative effects; $F_{X}\left(n-1\right)$ is the corresponding instantaneous cutting force at the previous step; and $\alpha \in \left(0,1\right)$ is the inertia coefficient.

## 3. Materials and Methods

### 3.1. Construction of the Sawing Platform

**Figure 10:**
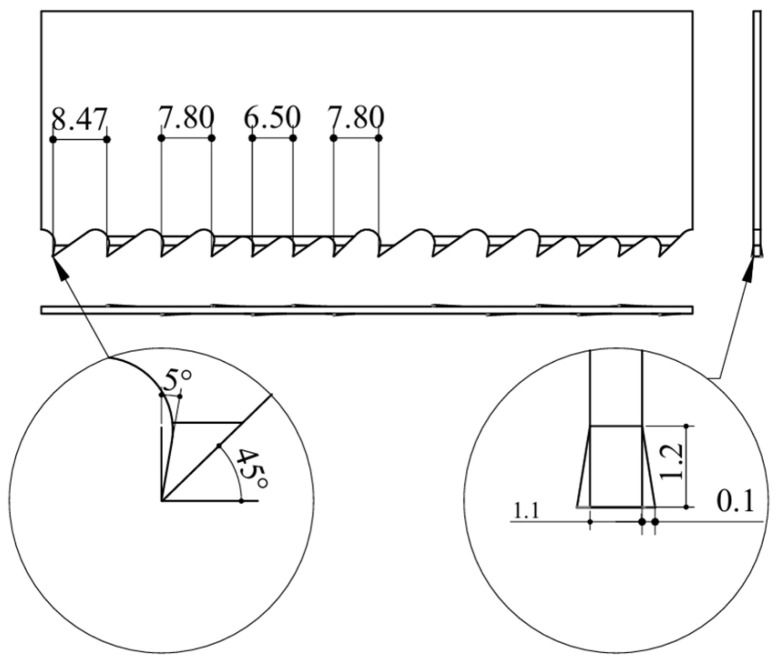
Schematic illustration of the band saw blade tooth geometry (all dimensions are in mm).

Experimental validation of the proposed ultrasonic vibration-assisted band sawing method and cutting force prediction model was conducted using a G4342 metal band saw (Zhejiang Chendiao Machinery Co., Lishui, China). The guide arm spacing was 770 mm. A T3/4 medium-pitch M42 band saw blade (Zhejiang Baoling Saw Industry Co., Ltd., Lishui, China) (4115 × 34 × 1.1 mm; Figure 10) was used to cut solid 304 stainless steel bars with a cross section of 30 × 50 mm. The material properties of the tool and workpiece are summarized in Table 4.

The band saw blade adopts a variable-pitch tooth design, in which straight teeth and set teeth are alternately arranged with alternating tooth sets. This complex cutting configuration provides a rigorous test of the robustness of the ultrasonic vibration-assisted band sawing method and enables validation of its effectiveness under complex cutting conditions, as well as the predictive accuracy of the variable-depth cutting force model. The pitch of the large teeth, small teeth, and transition teeth was 8.47 mm, 6.50 mm, and 7.80 mm, respectively. The tooth sequence consisted of three large teeth followed by four small teeth, and within one tooth period, one straight tooth corresponded to six set teeth. The rake angle was 5°, the tooth set was 0.1 mm, the blade thickness was 1.1 mm, and the cutting edge length was 1.2 mm. Figure 10 shows the geometric features of a single tooth period of the band saw blade with a length of 50.8 mm.

**Figure 11:**
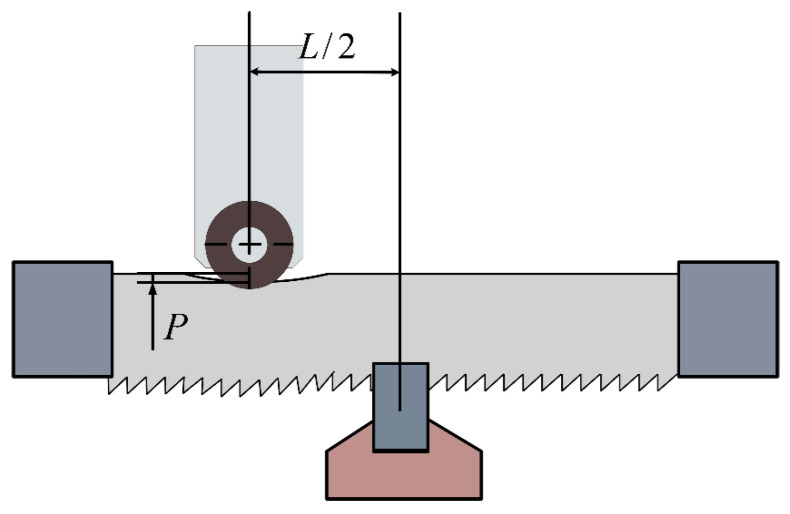
Schematic illustration of ultrasonic vibration-assisted sawing ( P denotes the preload).

When an ultrasonic excitation with an amplitude of m mm is applied along the feed direction of the band saw at a distance of mm from the workpiece center, the feed velocity is much smaller than the sawing speed $v_{c}$ and is negligible compared with the instantaneous feed component induced by the ultrasonic excitation. Consequently, the band saw blade primarily exhibits a periodic vibration response during sawing. The corresponding sawing configuration is illustrated in Figure 11, where a periodic vibration-induced response function of the band saw blade is observed above the workpiece.

### 3.2. Design of the Ultrasonic Vibration Excitation Device

The ultrasonic excitation system used in this study consists of two core modules: an ultrasonic device mounting module and an ultrasonic excitation generation module. Based on a systematic analysis of existing metal band saw configurations, a simplified in-house ultrasonic vibration-assisted sawing device was developed. The device applies lateral ultrasonic excitation to the back edge of the band saw blade, inducing periodic vibration while closely replicating the guide arm support conditions.

**Figure 12:**
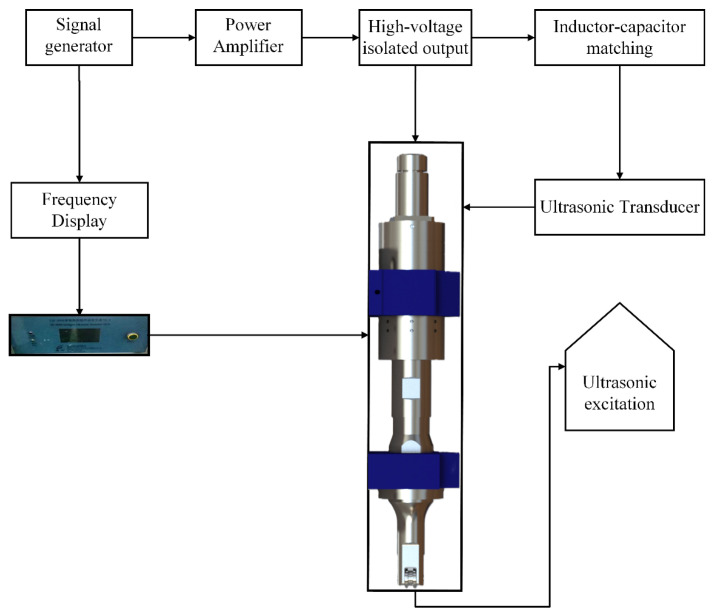
Operating principle of the ultrasonic excitation system.

The ultrasonic excitation system is mounted on the band saw crossbeam, with the excitation generation module integrated into the mounting structure. The system consists of a signal generator and an ultrasonic transducer (Figure 12). The generator converts AC power into a pulsed DC signal, which is transformed into mechanical vibration by the transducer and delivered to the back edge of the band saw blade via a horn. The horn employs an H-shaped wheel design to emulate the guide wheel, constrain lateral blade deflection, and provide a rolling, simply supported boundary condition. High-frequency vibrations generated by the transducer are transmitted through the horn and guide wheel and applied along the blade feed direction.

Within a specified length range, the device creates an unsupported band saw cutting condition to evaluate the effect of ultrasonic excitation on the sawing process. The specifications of the ultrasonic generator are summarized in Table 5.

**Figure 13:**
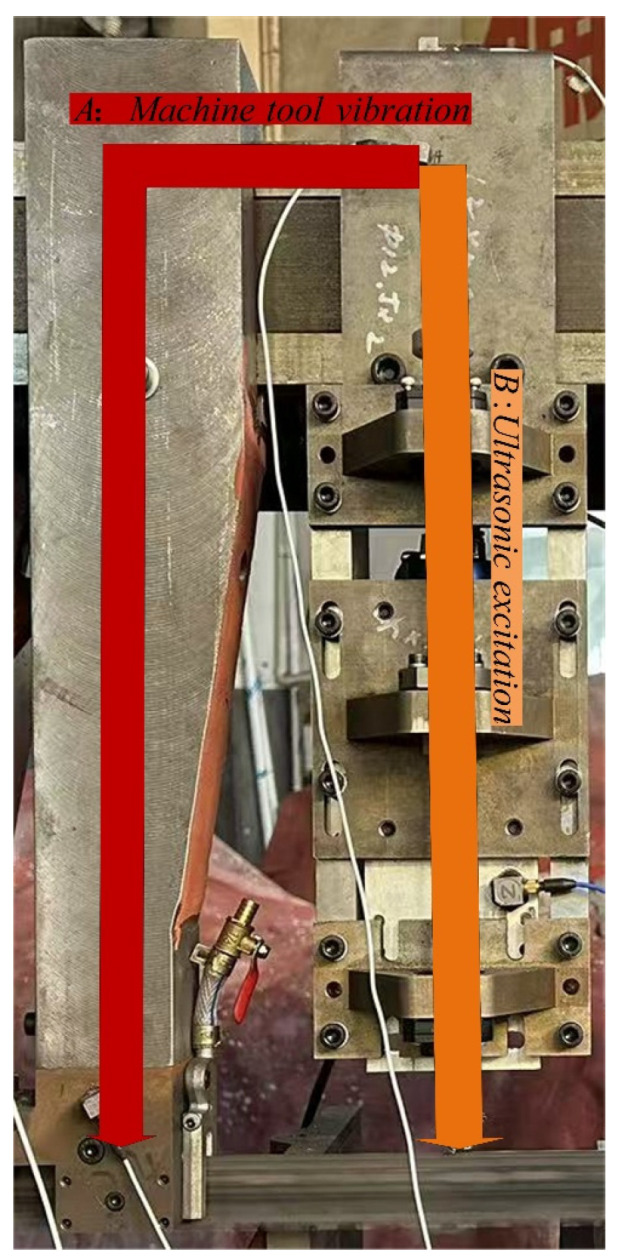
Schematic illustration of the vibration transmission path.

In the present experiment, a guide-arm-like support structure was installed at the L/2 position, where a guide wheel directly contacts the band saw blade and is integrated with an ultrasonic generator to enable direct excitation of the guide wheel. By applying a downward preload to the support structure, the transmission efficiency of the vibration excitation to the band saw blade is significantly enhanced under the blade tension. The transmission path of the ultrasonic excitation vibration is illustrated by path B in Figure 13.

### 3.3. Test Design

**Figure 14:**
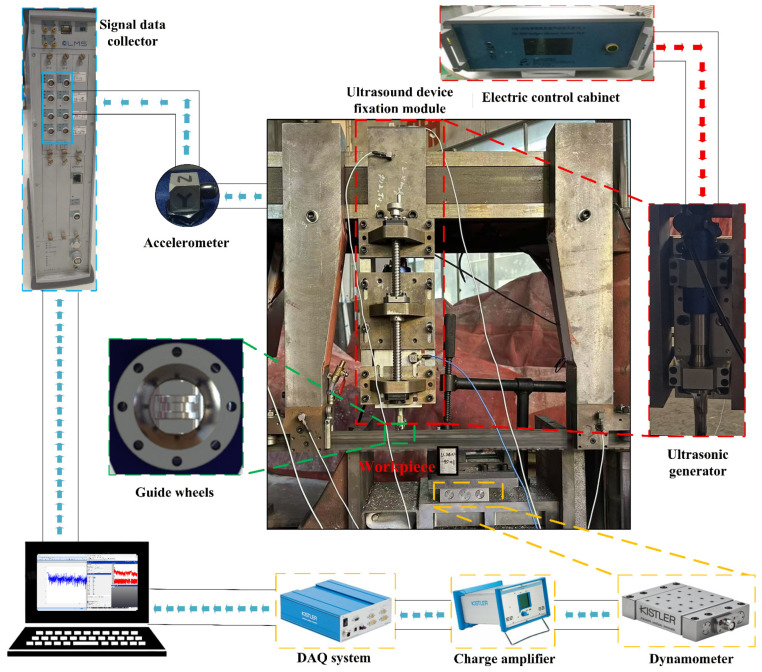
Vibration and force signal acquisition system. Red arrows indicate the transmission paths of vibration excitation, while blue arrows represent those of acquired signal transmission.

As shown in Figure 14, the ultrasonic vibration-assisted band sawing process was evaluated primarily in terms of cutting force and workpiece surface quality to assess the accuracy of the variable-depth cutting force prediction model and the process improvement achieved by ultrasonic assistance. Contact accelerometers were installed at key locations, including the band saw guide arm and the horn base plate, and vibration signals were acquired via a data acquisition system to evaluate the effectiveness of the ultrasonic excitation and the actual vibration response of the band saw blade [22]. A compression-type force sensor was installed beneath the workpiece, together with a charge amplifier and a data acquisition unit, to record the cutting force signals. The operating parameters of the vibration measurement and cutting force acquisition systems are summarized in Table 6 and Table 7.

**Figure 15:**
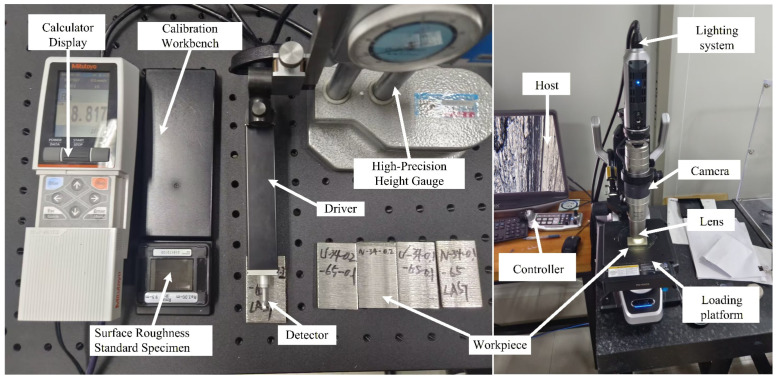
Sampling and analysis system for the machined workpiece surface.

As shown in Figure 15, the machined surface of the workpiece was further sampled and analyzed after cutting. Surface damage was examined using a depth-of-field surface imaging system. Surface profiles were measured with a contact probe, and profile data were acquired using a stylus-type surface roughness tester to determine surface roughness parameters and evaluate the surface quality of the workpiece.

## 4. Results and Discussion

### 4.1. Cutting Force Prediction

To validate the proposed prediction model, a series of sawing experiments was conducted on 304 stainless steel workpieces. The workpiece material was prepared through forging and high-temperature melt casting combined with a liquid–solid phase transformation process. A single-factor experimental design was adopted, with the variable ranges determined based on conventional sawing parameters for stainless steel. Owing to the introduction of external ultrasonic excitation, the feed rate was set to 0.1–0.4 mm/s.

In conventional band sawing, stepped speed regulation requires blade replacement, and the slip between the blade and wheels makes the actual sawing speed difficult to determine accurately. Therefore, the sawing speed was fixed at 34 m/min to ensure consistent cutting conditions, and ultrasonic vibration cutting force responses were analyzed under preload values ranging from 0 to 0.5 mm.

Considering the effects of preload and feed rate, a corrected sawing speed model is established and can be expressed as Equation:

(27) $${v_{c}}^{\prime }=v_{c}\cdot \left(\alpha_{c}P_{L}+\beta_{c}v_{f}+\gamma_{c}\right)$$

where $\alpha_{c}$, $\beta_{c}$, and $\gamma_{c}$ denote slip coefficients introduced to account for blade–wheel interaction.

Based on the above parameter settings, a total of 36 experimental conditions were designed by varying the sawing speed and preload. To reduce experimental uncertainty, each condition was repeated three times.

**Figure 16:**
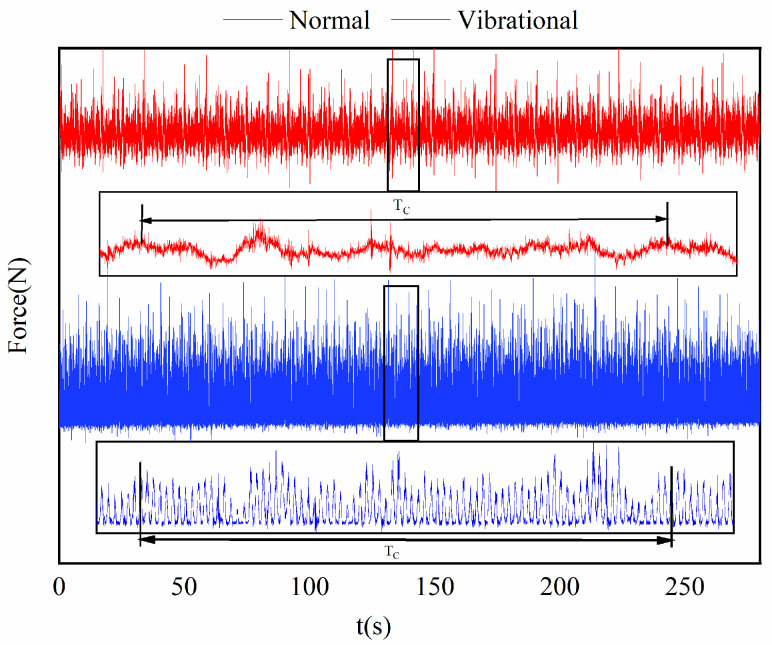
Cutting force measurement results (Normal: conventional sawing; Vibrational: ultrasonic vibration-assisted sawing).

Figure 16 illustrates the time evolution of the main cutting force of the band saw blade. At a preload of 0 mm, the band saw operates under conventional sawing conditions, and the cutting force exhibits periodic fluctuations with a period approximately equal to one blade revolution. This periodic behavior mainly arises when the welded joint of the blade passes through the workpiece, causing a sudden increase in kerf width and cutting cross-sectional area, which leads to pronounced variations in the main cutting force. At smaller time scales, random vibrations are superimposed, resulting in a relatively wide fluctuation band of the cutting force signal.

When the ultrasonic excitation preload reaches 0.1 mm or higher, the cutting force in ultrasonic sawing exhibits pronounced periodic fluctuations at much smaller time scales. Therefore, a micro-period cutting force prediction model based on variable-depth sawing is required to accurately capture the dynamic force characteristics of ultrasonic vibration-assisted band sawing.

#### 4.1.1. Parameter Fitting

**Figure 17:**
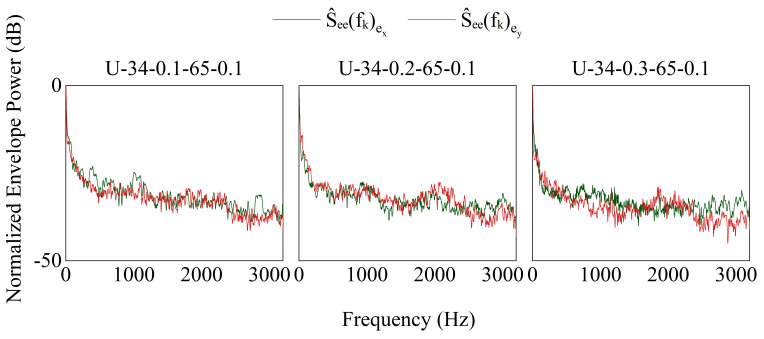
Vibration envelope spectrum.

**Figure 18:**
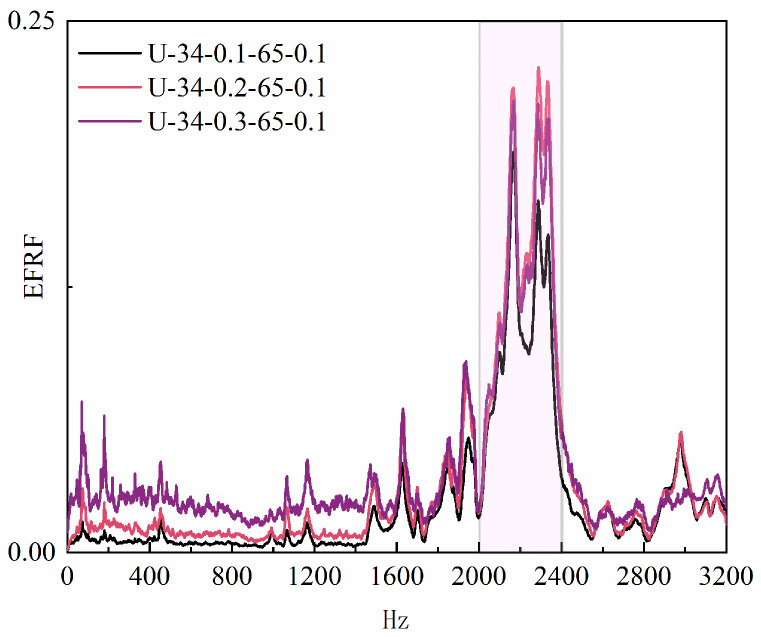
Vibration frequency response.

To determine the actual vibration frequency f and tension σ of the band saw blade, the input ultrasonic excitation signal was further corrected based on vibration transmission characteristics to identify the minimum envelope frequency of the blade vibration. By analyzing the self-envelope and cross-envelope spectra of the vibration input and actual band saw blade vibration signals (Figure 17), the envelope power spectral shapes and trends were consistent, validating the stable and high-fidelity excitation effect of contact ultrasonic vibration in the band saw system [23]. Finally, the frequency response function analysis (Figure 18) shows that the minimum vibration frequency of the band saw blade is approximately 2200 Hz, and the blade tension is around 180 MPa.

To determine the optimal initial values for cutting pressure ratio $k_{t}$ and linear deviation $k_{\varepsilon }$ and obtain the global optimum, the cutting pressure ratio was initially set to $10^{2}$ N/mm^2^ based on the material properties listed in Table 5. Based on this, MATLAB 2019 and Origin data analysis tools were used to perform regression fitting of the experimental data. The fitted data cover four feed speeds (0.1, 0.2, 0.3, 0.4 mm/s) and three preload values (0.1, 0.3, 0.5 mm), comprising 12 sets of experimental data. The Levenberg–Marquardt optimization algorithm was used to iteratively converge and determine the fitting parameters in the regression equation.

The fitting results show that the cutting pressure ratio $k_{t}=100.67$ N/mm^2^ and the linear deviation $k_{\varepsilon }$ = −2.03 N. From this, the transient cutting force calculation formula is derived as follows:

(28) $$F_{X}=100.67\cdot \sum_{i=m}^{n}A_{i}^{\prime }-2.03$$

Additionally, multiple linear regression using Origin yields the following values from Equation (27): $\alpha_{c}$ = −0.5832, $\beta_{c}$ = −1.070, and $\gamma_{c}$ = 1.046. Therefore, the sawing speed expression considering slip is given by:

(29) $${v_{c}}^{\prime }=v_{c}\cdot \left(-0.5832P_{L}-1.070v_{f}+1.046\right)$$

#### 4.1.2. Prediction Model Validation

**Figure 19:**
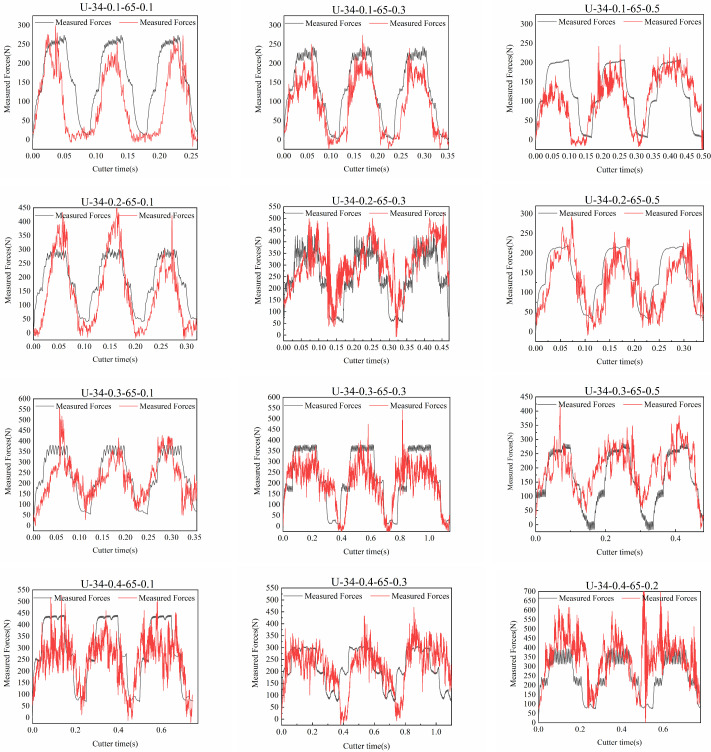
Comparison of the variable-depth force prediction model and experimental data.

As shown in Figure 19, the measured cutting force curve is compared with the predicted dynamic cutting force curve based on the variable-depth model. Under different operating conditions, the experimental cutting force curve closely matches the trend of the theoretical model. Under feed speeds of 0.1–0.4 mm/s and preload values of 0.1–0.5 mm, the periodicity and amplitude of and instantaneous changes in the cutting force closely match the predicted model, validating the accuracy of the variable-depth dynamic cutting force model. Furthermore, a preliminary dynamic cutting force prediction framework for vibration-assisted sawing has been established.

Under a preload of 0.3 mm and certain higher feed speeds, the amplitude and trend of the measured curve remain highly consistent, with periodic differences and minimum value errors within acceptable limits, further supporting the model’s reliability and applicability.

To quantitatively evaluate the accuracy of the prediction model, the dynamic cutting force error rate ( $e_{r}$) is used. Here, $e_{r}$ is defined as the ratio of the difference between the measured value and the theoretical prediction to the measured value:

(30) $$S_{I}=\frac{\sum_{i=1}^{n}\frac{F_{i}-{\hat{F}}_{i}}{F_{i}}}{n-1}$$

As shown in Table 8, the preliminary dynamic cutting force prediction model performs well under various conditions: the error in single dynamic cutting force is within 50 N, the maximum dynamic cutting force error rate is 42.3%, and in most conditions, the error rate is below 20%. The overall average error rate of dynamic cutting force prediction is 5.44%.

When both the feed rate and preload are high—for instance, a feed rate of 0.4 mm/s and a preload of 0.5 mm—the prediction model still functions, but a relatively high prediction error is observed. Analysis indicates that under this condition, the cutting force increases substantially compared to other conditions, with peak values generally exceeding 500 N. This reflects a significant increase in the single-tooth cutting volume. The increased cutting volume exacerbates band saw slippage, while the high preload prevents the teeth from effectively performing intermittent cutting at entry and exit. As a result, the cutting pattern deviates from the theoretical model, further amplifying the prediction error. Therefore, when the feed rate or preload is relatively high, the accuracy of the prediction model is partially reduced.

When both the feed rate and preload are very low—for example, a feed rate of 0.1 mm/s and a preload of 0.1 mm—the prediction model error also increases. The model demonstrates accurate periodic predictions, and the band saw cutting speed does not exhibit significant periodic prediction errors due to excessive slippage. However, peak value predictions are clearly inaccurate, which is consistent with the previously noted detachment between the vibration excitation and the band saw, resulting in poor vibration transmission. Therefore, it is preliminarily inferred that the large prediction error in cutting force is caused by inadequate vibration contact, leading to poor vibration transmission.

The prediction model is effective only under commonly used processing parameters for hard-to-machine materials. Prediction errors increase when vibration transmission efficiency is low or when band saw slippage occurs.

Considering unexpected variations in cutting conditions and manual deviations in tension adjustment during actual band saw operations, prediction errors remain within acceptable limits under other commonly used processing parameters for hard-to-cut materials. This confirms the accuracy and reliability of the dynamic cutting force prediction model.

### 4.2. Analysis of Vibration-Assisted Sawing Performance

#### 4.2.1. Cutting Force Optimization

In conventional sawing, the periodicity of the cutting force curve is primarily mapped to the band saw’s rotational cycle. The large fluctuations reflect cutting force variations caused by the band saw’s rotation, while short-term random vibrations generated when the flexible band compresses the workpiece are superimposed on the periodic fluctuations [18]. Ultrasonic vibration-assisted sawing achieves intermittent cutting and introduces short-term periodic tool retraction, effectively suppressing the short-term random vibrations caused by flexible band compression during cutting.

Based on common sawing parameters for 304 stainless steel, the experimental variable ranges were determined: sawing speed of 22–51 m/min, feed rate of 0.1–0.5 mm/s, and vibration preload of 0.1–0.5 mm, with a total of 216 experimental conditions designed. To reduce experimental error, each experimental condition was repeated three times. This study focuses on analyzing the sawing performance at a sawing speed of 34 m/min, applying ultrasonic vibration excitation to the tool at 65 cm from the workpiece center. The effect of vibration-assisted intermittent cutting on cutting force is quantified, and the main cutting force in band saw cyclic cutting is discussed as an example.

**Figure 20:**
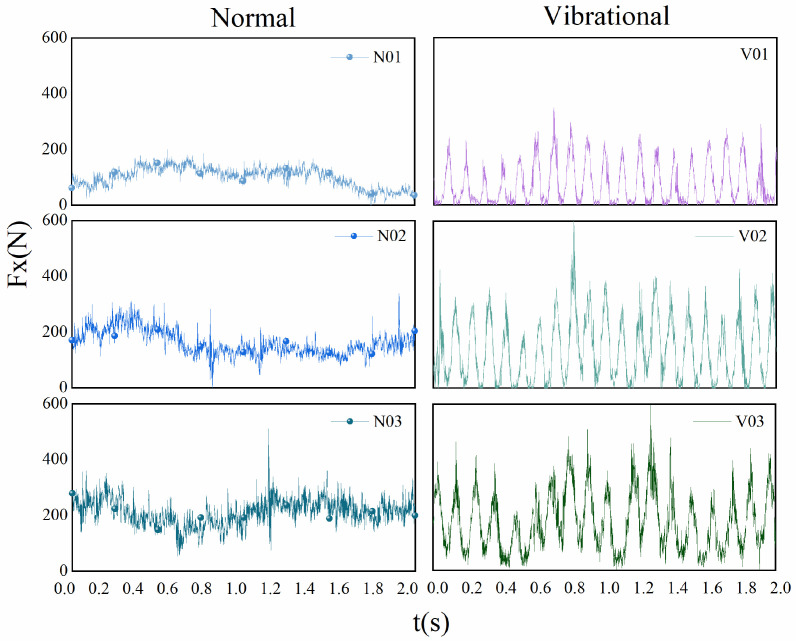
Comparison of the main cutting force between conventional sawing and ultrasonic vibration-assisted sawing. (N01, N02, N03 represent the main cutting force curves for conventional sawing at feed speeds of 0.1–0.3 mm/s, while V01, V02, V03 represent the main cutting force curves for ultrasonic vibration-assisted sawing under the same conditions.).

As shown in Figure 20, using a feed rate of 0.1 mm/s and a sawing speed of 34 m/min as an example, a single-factor control method was applied to conduct comparative experiments under different preload conditions, analyzing the effect of ultrasonic vibration on the fluctuation characteristics of cutting force. Preliminary experimental results indicate that when ultrasonic excitation is applied to the band saw, the original cutting and feed force curves undergo significant changes, resulting in a new sawing mode. While changing the main cutting and feed force curves, the effect on the lateral force is minimal.

In the conventional sawing process with N01, N02, and N03, the cutting force curve lacks distinct periodicity under short-term cutting conditions and is accompanied by significant random noise. Under the same sawing speed and feed rate conditions, the V01, V02, and V03 curves with ultrasonic excitation exhibit distinct short-term periodicity. Compared to conventional sawing, ultrasonic excitation shortens the cutting force period to approximately 0.2 s and significantly suppresses random vibrations caused by flexible band compression, resulting in a smoother and more stable cutting force curve.

When the ultrasonic excitation is applied at a position 65 cm from the workpiece center, and the sawing speed is 34 m/min, the optimization effect of vibration-assisted intermittent sawing on the average cutting force and average feed force is systematically studied for different feed rates and vibration preload values. During the experiments, the average cutting force and average feed force under each condition were recorded, and the results are summarized in Table 9, Table 10 and Table 11.

**Figure 21:**
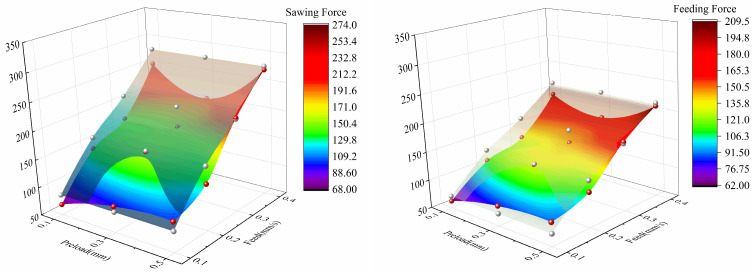
Comparison heatmap of ultrasonic vibration-assisted cutting force.

As shown in Figure 21, under operating conditions with feed speeds of 0.1–0.4 mm/s and preload values of 0.1–0.5 mm, ultrasonic vibration-assisted intermittent sawing significantly reduced the cutting force and feed force in certain conditions, thereby improving the sawing performance. When the preload is 0.1 mm, both the cutting force and feed force are significantly improved across the entire feed speed range of 0.1–0.4 mm/s. Under the condition of a preload of 0.3 mm, the improvement in sawing performance is more pronounced at higher feed speeds, such as 0.4 mm/s.

**Figure 22:**
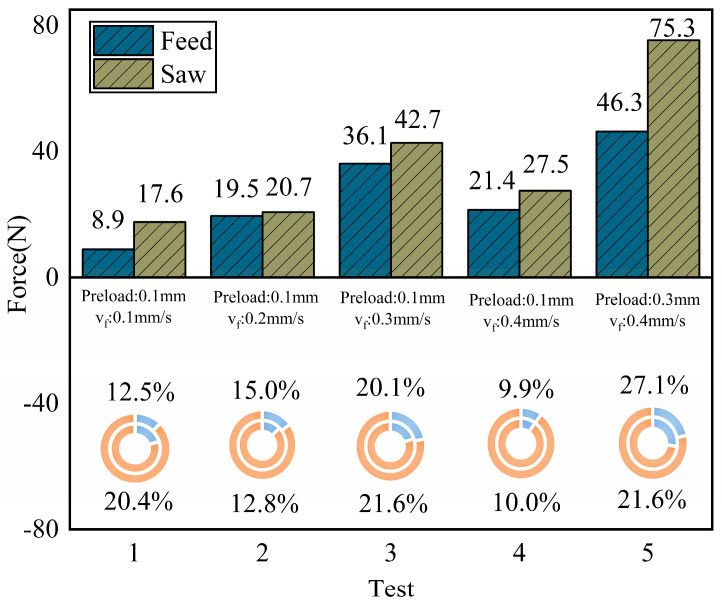
Optimization effect of ultrasonic vibration-assisted sawing on cutting force (upper part: reduction in force, lower part: reduction ratio relative to cutting force).

As shown in Figure 22, ultrasonic vibration-assisted sawing significantly optimizes both cutting force and feed force in certain conditions, with improvements typically reaching around 10%, and the improvement trend of feed force is similar to that of cutting force. Specifically, when the preload is 0.1 mm, for the conditions with feed speeds of 0.1–0.4 mm/s, the feed force decreases by 20.4%, 12.8%, 21.6%, and 10.0%, respectively, while the cutting force decreases by 12.5%, 15.0%, 20.1%, and 9.9%, respectively. When the preload is 0.2 mm, for the condition with a feed speed of 0.4 mm/s, the feed force decreases by 21.6%, and the cutting force decreases by 27.1%, indicating that preload and feed speed jointly affect the optimization effect of vibration-assisted sawing.

#### 4.2.2. Optimization of Workpiece Surface Washboard Phenomenon

In conventional sawing, overlapping tooth cutting paths and mismatched cutting parameters can lead to the washboard effect on the workpiece surface [4]. This results in periodic high-low ridges resembling a washboard pattern. This not only reduces surface finish and subsequent processing accuracy but also leads to crack formation, damaging the internal material structure, increasing material consumption, and raising processing difficulty, becoming a technical challenge in practical machining. In contrast, ultrasonic vibration-assisted sawing demonstrates significant advantages in improving the washboard effect and reducing cracking, effectively enhancing surface quality. In this experiment, a KEYENCE ultra-depth 3D microscopy system (KEYENCE CORPORATION, Osaka, Japan) was used to analyze and compare the microscopic surface morphology of workpieces from ultrasonic vibration-assisted sawing and conventional sawing. The results, shown in Table 12, indicate that ultrasonic vibration-assisted sawing significantly outperforms conventional sawing: surface cracks are greatly reduced, and surface smoothness is notably improved. The optimization effect is most significant under preload values of 0.1 mm and 0.3 mm, while the improvement slightly decreases when the preload is 0.5 mm.

The experiment further utilized a contact surface morphology measurement system, Mitutoyo SurfTest SJ (Mitutoyo Corporation, Kawasaki-shi, Kanagawa, Japan), along with the SJ-210 surface roughness tester, to measure the profile of the workpiece cutting surface along the direction perpendicular to the washboard effect. Combining the surface profile analysis along the feed direction allows for a more intuitive assessment of the optimization effect of ultrasonic vibration-assisted sawing on surface quality [24].

**Figure 23:**
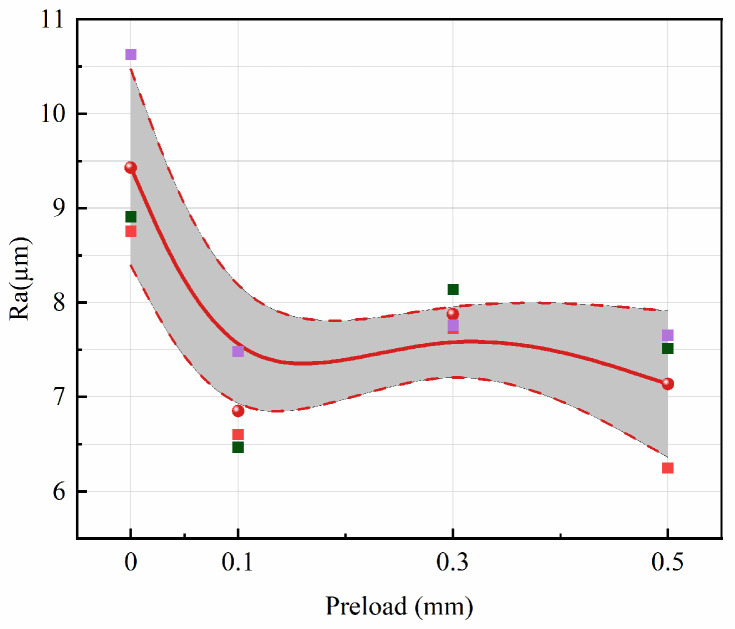
Effect of preload on surface optimization. The shaded region denotes the measured surface roughness range, bounded by the dashed lines.

The optimization effect of ultrasonic excitation preload on the surface quality of the workpiece along the feed direction was quantitatively analyzed using the arithmetic average roughness $R_{a}$. As shown in Figure 23, under the same conditions, ultrasonic excitation with preload values of 0.1–0.3 mm reduced the surface roughness $R_{a}$ from approximately 9.5 µm to 7.5 µm. Additionally, based on the analysis in Figure 22, ultrasonic vibration-assisted sawing achieves consistent optimization at a preload of 0.1 mm. Therefore, 0.1 mm preload is selected as the default condition for ultrasonic excitation and compared with the corresponding conventional sawing conditions.

**Figure 24:**
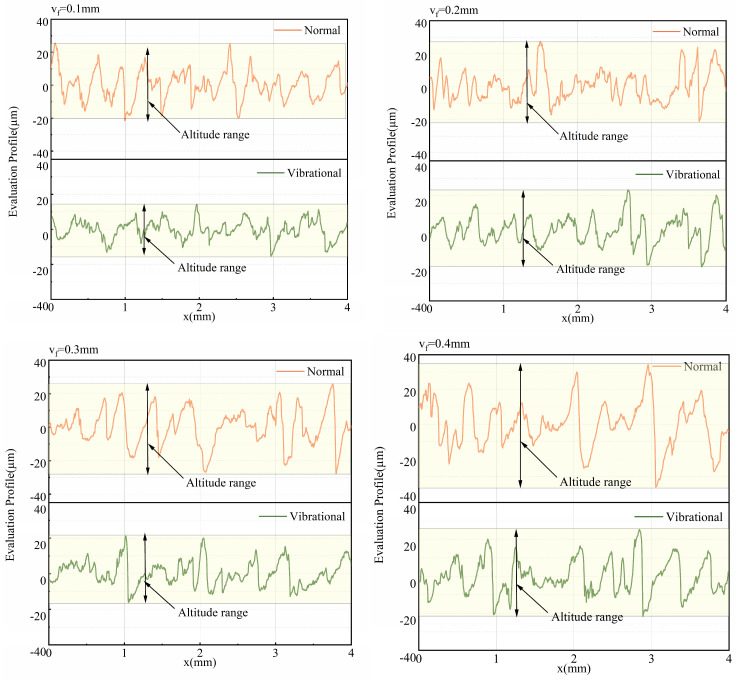
Comparison of workpiece surface profile (conventional sawing vs. ultrasonic vibration-assisted sawing).

Figure 24 shows the comparison of the workpiece surface profile along the feed direction for 304 stainless steel under ultrasonic vibration-assisted sawing and conventional sawing conditions (feed speed 0.1–0.4 mm/s). The results show that the surface profile range for ultrasonic vibration-assisted sawing is significantly smaller than that of conventional sawing, with the height difference in the surface profile being approximately half of that in conventional sawing. Under these conditions, ultrasonic vibration-assisted sawing not only reduces cutting force but also alleviates the issue of excessively deep cutting marks from individual teeth caused by flexible band compression, thus mitigating the washboard effect and improving surface quality [4].

**Figure 25:**
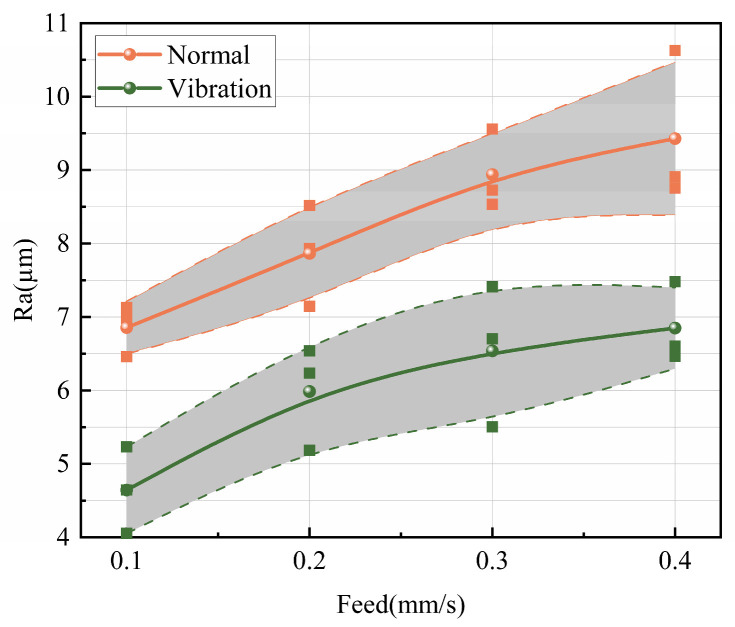
Surface quality optimization in ultrasonic vibration-assisted sawing. The shaded region denotes the measured surface roughness range, bounded by the dashed lines.

As shown in Figure 25, after introducing vibration excitation, the surface roughness $R_{a}$ of the workpiece in ultrasonic vibration-assisted sawing significantly decreases. In intermittent sawing, the band saw achieves better surface roughness compared to conventional sawing (with a preload of 0 mm), effectively improving the workpiece surface washboard effect. When the feed rate and sawing speed are constant, vibration excitation can reduce roughness by approximately 21% compared to conventional sawing under different feed speeds. Overall, ultrasonic vibration-assisted sawing demonstrates significant effectiveness in improving the workpiece washboard effect.

In summary, the roughness analysis above demonstrates the optimization effect of ultrasonic vibration-assisted sawing under a wide range of sawing conditions. This method significantly reduces the surface roughness along the feed direction of the workpiece, lowers the risk of crack formation, and decreases the material removal and surface treatment consumables required for subsequent finishing, thus improving processing efficiency and workpiece surface quality.

## 5. Conclusions

To optimize the sawing process, improve processing efficiency and quality, and achieve precise control of dynamic cutting forces, this study investigates ultrasonic vibration-assisted band sawing optimization and dynamic cutting force prediction analysis. Through sawing mechanism analysis, this study combines the cutting force prediction model with the string vibration equation to establish a dynamic cutting force model suitable for variable-depth cutting. Comparative experiments of conventional and ultrasonic vibration-assisted sawing were conducted based on a metal band saw, focusing on validating the consistency between the time-varying cutting force signals and the prediction model. Additionally, by combining experimental data, the effects of ultrasonic vibration-assisted sawing on cutting force optimization and workpiece surface defect improvement were systematically analyzed. The main conclusions are as follows:

1. Based on the dynamic cutting depth analyzed through the string vibration equation and considering the cross-sectional effects of adjacent teeth, the dynamic cutting section error was corrected, and a dynamic cutting force prediction model suitable for variable-depth sawing was developed. In experimental validation, the model achieved an average dynamic cutting force error rate of 5.44%, preliminarily proving its predictive accuracy.
2. Experimental results show that ultrasonic vibration-assisted sawing significantly reduces both cutting force and feed force at a preload of 0.1 mm, with reductions typically exceeding 10%. Under preload values of 0.3 mm and 0.5 mm, the cutting force also shows significant optimization in some conditions.
3. To verify the improvement effect of optimized cutting force conditions on workpiece surface quality and subsequent processing stages, this study analyzes the washboard effect in conventional sawing. Based on the surface roughness in the feed direction, the optimized cutting force conditions show that ultrasonic vibration-assisted sawing effectively reduces the washboard effect, decreases workpiece cracks, and improves surface quality, with line roughness reduced by approximately 21% compared to conventional sawing.

Ultrasonic vibration-assisted sawing, as an innovative sawing method, fills the band gap in saw vibration-assisted sawing technology and provides significant guidance for sawing process optimization and cutting force improvement. This study systematically analyzes the mechanism of ultrasonic vibration-assisted sawing and develops a dynamic cutting force prediction model, providing a theoretical foundation for ultrasonic vibration cutting force prediction. The model’s accuracy is validated through comparative sawing experiments, and the optimization effect of ultrasonic vibration-assisted sawing is evaluated by combining cutting force and workpiece surface quality. Providing a reliable basis for improving ultrasonic vibration-assisted sawing technology.

## 6. Patents

CN118768645A, ultrasonic vibration auxiliary sawing device.

## Figures and Tables

**Table 1:** Analytical solutions of the vibration equation for different excitation locations.

Excitation Location	Boundary Conditions	Solution to the Vibration Equation
Upstream	$\left\{\begin{array}{l} x=\frac{L+L_{0}}{2}\\ y=0 \end{array}\right.$ $\left\{\begin{array}{l} x=0\\ y=m\sin\left(\omega t\right)=\frac{m}{2i}\left[\exp\left(i\omega t\right)-\exp\left(-i\omega t\right)\right] \end{array}\right.$	$y_{1}\left(x,t\right)=-mi\frac{\sin\left[\omega \left(\frac{L+L_{0}}{2}-x\right)/c^{\prime }\right]}{\sin\left(\omega \frac{L+L_{0}}{2}/c^{\prime }\right)}\sin\left[\omega \left(t-\frac{x}{v^{\prime }}\right)\right],0<x<\frac{L+L_{0}}{2}$
Downstream	$\left\{\begin{array}{l} x=-\frac{L_{0}-L}{2}\\ y=0 \end{array}\right.$ $\left\{\begin{array}{l} x=L\\ y=m\sin\left(\omega t\right)=\frac{m}{2i}\left[\exp\left(i\omega t\right)-\exp\left(-i\omega t\right)\right] \end{array}\right.$	$y_{2}\left(x,t\right)=-mi\frac{\sin\left[\omega \left(x+\frac{L_{0}-L}{2}\right)/c^{\prime }\right]}{\sin\left(\omega \frac{L+L_{0}}{2}/c^{\prime }\right)}\sin\left[\omega \left(t-\frac{L-x}{v^{\prime }}\right)\right],-\frac{L_{0}-L}{2}<x<L$

**Table 2:** Interference cross-section equations.

Interference Type	Cross-Section Equation
Skewed–straight interference	$A_{\delta ai}=\left\{\begin{array}{l} S_{\delta a1},0\leq \varepsilon_{dfi}df_{i}\left(t_{i}\right)\leq h_{k};\\ S_{\delta a2},h_{k}<\varepsilon_{dfi}df_{i}\left(t_{i}\right)\leq h_{j};\\ S_{\delta a3},h_{j}<\varepsilon_{dfi}df_{i}\left(t_{i}\right)\leq h_{j}+\left[d_{fi}\left(t_{i}\right)-b_{0}\tan\theta_{i}\sin\theta_{i}\right];\\ S_{\delta a4},h_{j}+\left[d_{fi}\left(t_{i}\right)-b_{0}\tan\theta_{i}\sin\theta_{i}\right]<\varepsilon_{dfi}df_{i}\left(t_{i}\right)\leq \delta_{i}\sin\theta_{i}+h_{l};\\ S_{\delta a5},\delta_{i}\sin\theta_{i}+h_{l}<\varepsilon_{dfi}df_{i}\left(t_{i}\right)\leq b_{0}\sin\theta_{i}+h_{l};\\ S_{\delta a6},b_{0}\sin\theta_{i}+h_{l}<\varepsilon_{dfi}df_{i}\left(t_{i}\right)\leq +\infty . \end{array}\right.$
Skewed–skewed interference	$A_{\delta bi}=\left\{\begin{array}{l} S_{\delta b1},0\leq df_{i}\left(t_{i}\right)\leq \left(a_{i}+a_{i-1}\right)\tan\theta_{i};\\ S_{\delta b2},\left(a_{i}+a_{i-1}\right)\tan\theta_{i}<df_{i}\left(t_{i}\right)\leq B_{1};\\ S_{\delta b3},B_{1}<df_{i}\left(t_{i}\right)\leq B_{2};\\ S_{\delta b4},B_{2}<df_{i}\left(t_{i}\right)\leq \varepsilon_{dfi}df_{i}\left(t_{i}\right)+a_{i-1}\left(\tan\theta_{i}+\tan\theta_{i-1}\right);\\ S_{\delta b5},\varepsilon_{dfi}df_{i}\left(t_{i}\right)+a_{i-1}\left(\tan\theta_{i}+\tan\theta_{i-1}\right)<df_{i}\left(t_{i}\right)\leq B_{3};\\ S_{\delta b6},B_{3}<df_{i}\left(t_{i}\right)<+\infty . \end{array}\right.$

**Table 3:** Summary of process-parameter expressions.

Process Parameters	Equations
$h_{k}$	$h_{k}=\delta_{i}\tan\theta_{i}-\left[d_{i}-\sqrt{{\left(\frac{1}{2}b_{0}\right)}^{2}+{d_{i}}^{2}}\cos\alpha \right]$
$h_{j}$	$h_{j}=\left(\delta_{i}+b_{\delta ai}\sin\theta_{i}\right)\sin\theta_{i}-\left(d_{i}-\frac{\delta_{i}+b_{\delta ai}}{\tan\alpha }\right)$
$h_{l}$	$h_{l}=df_{i}\left(t_{i}\right)-\left[d_{i-1}-\sqrt{{d_{i}}^{2}+{\left(\frac{1}{2}b_{0}\right)}^{2}}\cos\alpha \right]$
$S_{\delta a1}$	$S_{\delta a1}=df_{i}\left(t_{i}\right)\tan\theta_{i}\left[d_{i-1}+\frac{1}{2}df_{i}\left(t_{i}\right)-\frac{\delta_{i}+b_{\delta ai}}{\tan\alpha }\right]$
$S_{\delta a2}$	$S_{\delta a2}=S_{\delta a1}+\frac{{\left[df_{i}\left(t_{i}\right)-h_{k}\right]}^{2}}{2\tan\theta_{i}}$
$S_{\delta a3}$	$S_{\delta a3}=h_{j}\cdot \tan\theta_{i}\left(d_{i}-\frac{\delta_{i}+b_{\delta ai}}{\tan\alpha }+\frac{1}{2}h_{j}\right)+\frac{1}{2}\tan\theta_{i}{\left[b_{\delta ai}-\tan\theta_{i}\left(d_{i-1}-\frac{\delta_{i}+b_{\delta ai}}{\tan\theta_{i}}+h_{j}\right)\right]}^{2}+b_{\delta ai}\cdot \left(\varepsilon_{dfi}df_{i}\left(t_{i}\right)-h_{j}\right)$
$S_{\delta a4}$	$S_{\delta a4}=S_{\delta a3}+b_{\delta ai}\left[h_{j}-b_{0}\tan\theta_{i}\sin\theta_{i}+d_{i-1}-\frac{\delta_{i}+b_{\delta ai}}{\tan\alpha }\right]-\frac{1}{2}\tan\theta_{i}{\left[\varepsilon_{dfi}df_{i}\left(t_{i}\right)-df_{i}\left(t_{i}\right)+d_{i-1}-\frac{\delta_{i}+b_{\delta ai}}{\tan\alpha }\right]}^{2}$
$S_{\delta a5}$	$S_{\delta a5}=S_{\delta a4}-\frac{1}{2\tan\theta_{i}}{\left[\varepsilon_{dfi}df_{i}\left(t_{i}\right)-df_{i}\left(t_{i}\right)-\delta_{i}\tan\theta_{i}\right]}^{2}$
$S_{\delta a6}$	$S_{\delta a6}=S_{\delta a3}-b_{\delta ai}\cdot \left(\varepsilon_{dfi}df_{i}\left(t_{i}\right)-h_{j}\right)+b_{\delta ai}\left[df_{i}\left(t_{i}\right)-b_{0}\tan\theta_{i}\sin\theta_{i}\right]+b_{0}\cos\theta_{i}\cdot \left(b_{\delta ai}-\frac{1}{2}b_{0}\sin\theta_{i}\right)-\frac{{\left(b_{0}\sin\theta_{i}-\delta_{i}\right)}^{2}}{2\tan\theta_{i}}$
$B_{1}$	$B_{1}=\varepsilon_{dfi}df_{i}\left(t_{i}\right)\left(1-\cos^{2}\theta_{i-1}\right)+\sqrt{{d_{i-1}}^{2}+\frac{1}{4}{b_{0}}^{2}}\cos\alpha_{i-1}-\sqrt{{d_{i}}^{2}+\frac{1}{4}{b_{0}}^{2}}\cos\alpha_{i}+b_{0}\sin\theta_{i-1}+\left(b_{\delta bi}-b_{lai}\right)\sin\theta_{i}-\Delta d_{i}$
$B_{2}$	$B_{2}=\varepsilon_{dfi}df_{i}\left(t_{i}\right)+\sqrt{{d_{i-1}}^{2}+\frac{1}{4}{b_{0}}^{2}}\cos\alpha_{i-1}-\sqrt{{d_{i}}^{2}+\frac{1}{4}{b_{0}}^{2}}\cos\alpha_{i}+b_{0}\sin\theta_{i-1}+\left(b_{0}-a_{i}-a_{i-1}\right)\sin\theta_{i-1}-b_{0}\sin\theta_{i}-\Delta d_{i}$
$B_{3}$	$B_{3}=\varepsilon_{dfi}df_{i}\left(t_{i}\right)+\frac{b_{\delta i}-a_{i}}{\tan\theta_{i}}-a_{i}\tan\theta_{i}-b_{\delta i}\tan\theta_{i-1}.$
bt	$bt=b_{0}\tan\theta_{i}-\frac{a_{i}\tan\left(\theta_{i}+\theta_{i-1}\right)}{\cos\theta_{i}}$
$S_{\delta b1}$	$S_{\delta b1}=df_{i}\left(t_{i}\right)b_{\delta bi}-df_{i}\left(t_{i}\right)bt\sin\theta_{i}+\frac{1}{2}d{f^{2}}_{i}\left(t_{i}\right)\sin^{2}\theta_{i}+\frac{d{f^{2}}_{i}\left(t_{i}\right)\sin^{3}\theta_{i}\cos\theta }{\cos2\theta_{i}}$
q	$q=b_{0}-\frac{a_{i}}{\cos\theta_{i}}-\frac{a_{i-1}}{\cos\theta_{i-1}\cos\left(\theta_{i}+\theta_{i-1}\right)}-\frac{b_{lai}-b_{\delta bi}}{\cos\theta_{i}}$
$S_{\delta b3}$	$S_{\delta b3}=S_{\delta b2}-\frac{1}{2}q^{2}\frac{\cos2\theta_{i}}{\tan\theta_{i}}$
$S_{\delta b4}$	$S_{\delta b4}=S_{\delta b3}-\frac{{\left(df_{i}\left(t_{i}\right)-B_{2}\right)}^{2}}{2{\left(\cos\theta_{i}-\sin\theta_{i}\tan\theta_{i}\right)}^{2}\tan\left(\theta_{i}+\theta_{i-1}\right)}$
$S_{\delta b5}$	$S_{\delta b5}=b_{\delta bi}\varepsilon_{dfi}df_{i}\left(t_{i}\right)-{\left[\varepsilon_{dfi}df_{i}\left(t_{i}\right)-df_{i}\left(t_{i}\right)+\frac{b_{\delta i}-a_{i}}{\tan\theta_{i}}-a_{i}\tan\theta_{i}-b_{\delta i}\tan\theta_{i-1}\right]}^{2}\cdot \frac{\frac{b_{\delta i}-a_{i}}{\sin\theta_{i}}-\frac{a_{i}\tan\left(\theta_{i}+\theta_{i-1}\right)}{\cos\theta_{i}}}{\frac{b_{\delta i}-a_{i}}{\tan\theta_{i}}-a_{i}\tan\theta_{i}-b_{\delta i}\tan\theta_{i-1}}-\frac{1}{2}bt^{2}\cos2\theta_{i}\tan\theta_{i}$
$S_{\delta b6}$	$S_{\delta b6}=b_{\delta bi}\varepsilon_{dfi}df_{i}\left(t_{i}\right)-\frac{1}{2}bt^{2}\cos2\theta_{i}\tan\theta_{i}$

Table 3 provides supplementary explanations for the cross-sectional equations presented in Table 2, focusing on the cross-sectional areas and boundary conditions at different stages. All process variables are expressed in terms of band saw geometric parameters and cutting process parameters, facilitating the implementation of the dynamic cutting force prediction model using computational methods.

**Table 4:** Physical properties of M42 and 304.

Material	Density (g/cm^3^)	Young’s Modulus (GPa)	Melt Temperature (°C)	Specific Heat (J/(kg °C))	Heat Conductivity (W/(m °C))	Expansion Coefficient (×10^−6^/°C)
M42	8.16	220	1510	460	24	10.4
304	8.00	193	1450	500	16.2	17.3

**Table 5:** Ultrasonic Generator Parameters.

Device Model	Applicable Frequency/KHz	Applicable Capacitor/pf	Power Adjustment	Work Mode
TJS-3000	21	500–30,000	30–100%	Continuous

TJS-3000 (Hangzhou Chenrong Ultrasonic Equipment Co., Ltd., Hangzhou, China).

**Table 6:** Parameters of the vibration test equipment.

Equipment	Specifications	Performance
Mobile workstation	ROG Strix G614JV	None
Data Collection Frontend	SCADAS· III -305	Channel: 24 Maximum sampling frequency: 204.8 KHz
Software	LMS.Test.Lab7.0	Bandwidth: 0~25.6 KHz
Accelerometer	PCB 333B30	Sensitivity: (±10%) 100 mV/g Frequency Range (±5%): 0.5~3000 Hz
	PCB 356A02	Sensitivity: (±10%) 10 mV/g Frequency Range: (±5%) 1~10,000 Hz

ROG Strix G614JV (ASUS Computer Inc., Shanghai, China); SCADAS· III -305 (Siemens Ltd., Beijing, China); PCB 333B30 (KEYENCE (CHINA) Co., Ltd., Shanghai, China); PCB 356A02 (KEYENCE (CHINA) Co., Ltd.).

**Table 7:** Parameters of the force measurement equipment.

Equipment	Specifications	Performance
Mobile workstation	ROG Strix G614JV	None
DAQ	5697A	Channel: 28
		Sampling Rate: 1000 kS/s
Software	Dynaware 2825D-03	Resolution (per channel): 16-bit
Multiple-component force gauge	9129AA	Range: −10~10 kN
		Natural Frequency: 3.5~4.5 kHz

**Table 8:** Cutting force error rate (%).

$S_{I}$	Preload		
Feed Speed	0.1	0.3	0.5
0.1	41.2	33.7	23.0
0.2	19.3	−23.6	11.4
0.3	0. 47	9.54	−22.9
0.4	15.0	0.38	−42.3

**Table 9:** The force under different working conditions of normal (unit: N).

Force	Feed Speed			
	0.1 mm/s	0.2 mm/s	0.3 mm/s	0.4 mm/s
Feeding	71.22	130.1	166.8	214.8
Sawing	86.17	161.6	212.8	279.1

**Table 10:** The sawing force under different working conditions of vibrational (unit: N).

Preload	Feed Speed			
	0.1 mm/s	0.2 mm/s	0.3 mm/s	0.4 mm/s
0.1 mm	68.59	140.9	170.1	251.6
0.3 mm	96.11	159.2	176.5	203.8
0.5 mm	102.3	131.3	214.3	273.2

**Table 11:** The feeding force under different working conditions of vibrational (unit: N).

Preload	Feed Speed			
	0.1 mm/s	0.2 mm/s	0.3 mm/s	0.4 mm/s
0.1 mm	62.34	110.6	130.7	193.4
0.3 mm	84.63	128.5	144.1	168.5
0.5 mm	90.02	110.5	169.5	209.5

**Table 12:** Microscopic surface morphology scanning images of the workpiece.

Feed	0.1 mm/s	0.2 mm/s	0.3 mm/s	0.4 mm/s
Magnification	×30.0			
None	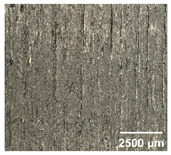	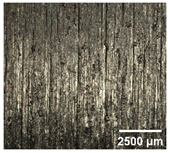	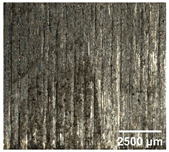	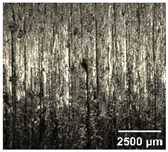
0.1 mm	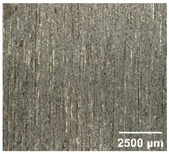	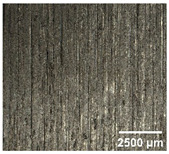	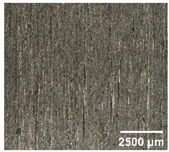	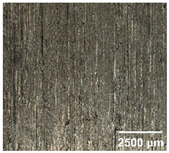
0.3 mm	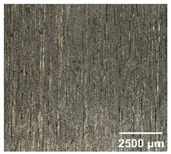	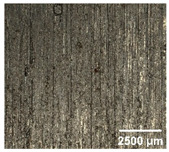	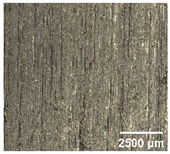	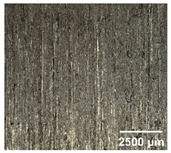
0.5 mm	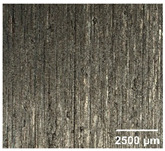	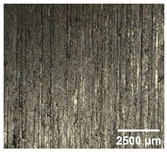	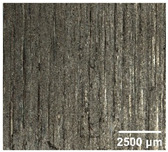	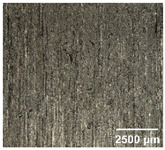

## Data Availability

The original contributions presented in this study are included in the article. Further inquiries can be directed to the corresponding author.
